# Effects of mating-type ratio imbalance on the degeneration of *Cordyceps militaris* subculture and preventative measures

**DOI:** 10.7717/peerj.17648

**Published:** 2024-07-09

**Authors:** Xin Wang, Xiu’E Li, Wenxu Qiu, Fangping Sa, Yetong Feng, Yupeng Ge, Shude Yang, Yu Liu, Jinzhong Xie, Wei Zhang, Weihuan Li, Xianhao Cheng

**Affiliations:** 1 School of Agriculture, Ludong University, Yantai, Shandong, China; 2 Research Institute of Subtropical Forestry, Chinese Academy of Forestry, Hangzhou, Zhejiang, China

**Keywords:** *Cordyceps militaris*, Strain degeneration, Mating type genes, MAT1-1, Disproportion

## Abstract

The rapid degeneration of *Cordyceps militaris* strains during subculture represents a bottleneck problem that affects production stability. This study explored the mechanism underlying this degeneration in three production and three wild-type strains of *Cordyceps militaris*, isolating single-conidium strains from each. The effects of subculturing on fructification in both original and single mating-type strains were compared. Changes in the ratio of the two mating types were analyzed in both original and degenerated strains. Based on these findings, the two mating strains were paired in different ratios to determine their effects on fruiting. The resulting five strains were heterokaryotic strains with both MAT1-1 and MAT1-2 mating-type genes. Strain jb-2 was a single mating type (MAT1-1) mutant strain that produced stable fruiting bodies but failed to produce ascospores. It was found that the loss of or imbalance in mating types was the main reason for the rapid degeneration of fruiting traits during subculture and that this occurred randomly in the MAT1-1 and MAT1-2 types. The strains differed significantly in their stability during subculture. Fruiting was stable in the single mating-type Jb-2 strain, and the eleventh-generation fruited normally. There were differences in yield between the production and wild strains after inoculation with spawn containing different proportions of mating types. The production strain was more stable when inoculated with strains with mating-type ratios of 1:9 to 9:1 without affecting the yield. However, the yield of the wild-type strain xf-1 was positively correlated with the proportion of the MAT1-2 type, while the other two strains showed no correlations. Subculturing single mating-type mycelia separately and mixing them before production effectively mitigated degeneration during subculture. For *Cordyceps militaris* breeding, selecting strains containing both mating types, which are insensitive to the proportion of mating-type genes, enhanced stability in subculture and reduced the risk of mating-type loss. Direct breeding of specific single-mating type strains to induce fruiting is thus an effective breeding strategy.

## Introduction

Most cultivated fungi are basidiomycetes. In recent years, however, there has been a significant increase in the cultivation and production of edible fungi (*e.g.*, *Cordyceps militaris* and morels) belonging to the Ascomycota (Dong et al., 2016). However, both *Cordyceps militaris* and morels show poor stability on subculturing with rapid degeneration of the fruiting trait during ascomycete cultivation and production. This is a common technical problem encountered in the cultivation and production of ascomycetes.

*Cordyceps militaris* (L.) Fr Link belongs to the phylum Ascomycota, with the classification class Sordariomycetes, order Hypocreales, and family Cordycipitaceae, and represents the type species of the *Cordyceps* genus (Shrestha et al., 2012). *Cordyceps militaris* contains cordycepin, adenosine, cordycepic acid (mannitol), and Cordyceps polysaccharide and has antineoplastic, immunomodulatory, antioxidant, sedative-hypnotic, anti-inflammatory, and antibacterial properties (Reis et al., 2013; Yoon et al., 2015; Liu et al., 2017; Qin et al., 2019). Rabie (2022) found that cordycepin also has antiviral activities and was effective in treating COVID-19. *Cordyceps militaris* has been classified as a new resource food by the Ministry of Health of China in 2009 and was approved as a new food raw material in 2014, with bright market prospects (No. 3 Announcement of the Ministry of Health of the People’s Republic of China, 2009).

*Cordyceps militaris* is a typical dipolar heterothallic ascomycetes. Its heterokaryon contains two mating genes (MAT1-1and MAT1-2), with only two mating genes existing at the same time to complete the sexual life history (Gao, 2008), Single mating type strains either cannot form fruiting bodies or do not produce ascospore on the stroma (Shrestha et al., 2012; Shrestha, 2005). Studies have shown that MAT1-1 single-mating strains can form fruiting bodies, but do not produce ascospore (Wen, Li & Kang, 2012; Zheng, Xia & Xiao, 2011). The mating type of *Cordyceps militaris* is controlled by a pair of alleles (MAT1-1 and MAT1-2), which are the key regulatory factors controlling the mating of *Cordyceps militaris* and regulating the development of fruiting bodies (Kronstad & Staben, 1997). The mating site MAT1-1 consists of three adjacent genes encoding alpha-box protein MAT1-1-1 gene, containing HPG domain MAT1-1-2 gene and containing HMG (high mobility group) MAT1-1-3 gene. A genome-wide study of *Cordyceps militaris* showed that it did not contain the MAT1-1-3 gene (Zheng, Xia & Zhang, 2013). The mating site MAT1-2 only contains MAT1-2-1 gene , so the mating type of *Cordyceps militaris* can be determined by the presence of the mating-type MAT1-1 and MAT1-2 genes (Yokoyama, Yamagishi & Hara, 2003).

Despite the rapid increase in *Cordyceps militaris* production in China over the past decade, observations indicate that the fungus is unstable during subculture. Significant degeneration of *Cordyceps militaris* typically occurs after three to four generations (Yin, 2018), manifesting as a reduction in the fruiting body primordia, significant increases or decreases in sporulation, extended culture periods, reduced growth, or an absence of fruiting bodies, abnormal growth, reduced concentrations of both cordycepin and adenosine with significant reductions in carotenoid, and decreased cellulase and amylase activities, together with reduced expression of sex-related genes (Lin et al., 2010; Zhou et al., 2019; Chen et al., 2017; Sun et al., 2017a; Sun et al., 2017b; Yu, 2020; Wellham et al., 2021), all of which severely affect the stability of yield and production.

Several measures to counteract the reduced stability of *Cordyceps militaris* during subculture have been investigated, including reducing the number of generations, storage at 4 °C, strain rejuvenation, silkworm tieback, single ascospore mating hybridization of opposite mating type genes, and the isolation of ascospores. Growth media optimization has also been investigated by the addition of lysine and K^+^, Ca^2+^, and Zn^2+^ (Sun et al., 2017a; Sun et al., 2017b; Xia et al., 2012; Ma et al., 2017). However, none of these methods has been successful in eliminating the problem of *Cordyceps militaris* strain degeneration.

A change in karyotype is hypothesized to be the primary cause of *Cordyceps militaris* strain degeneration. However, whether this karyotypic change occurs in all strains remains unclear. This issue requires investigation to provide a solution for the effective production of *Cordyceps militaris*. The present study investigated a single mating-type strain of *Cordyceps militaris*, focusing on subculture impact, mating-type combinations, and fruiting to determine the primary causes of strain degeneration. Strategies to manage strain degeneration were also addressed.

## Materials & Methods

### Strains and media

#### Strains

Wild strains of *Cordyceps militaris*, specifically, xf-1 from Xifeng County, Liaoning Province, ms-1 from Mengshan Mountain, Shandong Province, and jyt-1 from Jingyuetan Park, Changchun City, were collected. Each *Cordyceps militaris* strain was identified by BLAST alignments against sequences in the GenBank database after DNA extraction and ITS sequence amplification. Strains xm-1, jb-1, and jb-2 were produced by Shandong Meiao Biological Engineering Co., Ltd. All strains were stored in the slant surface of the PDA medium (see below) in the strain storage center at the College of Agriculture, Ludong University, China.

#### Media

PDA medium: potato 200 g; agar 18 g; glucose 20 g; water 1000 mL; pH 5.5−6.5. Liquid seed medium: glucose 20 g; peptone 15 g; yeast powder 5 g; magnesium sulfate 1 g; potassium dihydrogen phosphate 2 g; VB1 0.1 g; water 1000 mL; pH 5.5−6.5. Fruiting-body culture medium: Oat 30 g, water 48 mL. Medium sterilization conditions: 121 °C; sterilization for 25 min.

### Methods

#### Verification of fruiting and identification of mating-type genes in strains

Verification of fruiting: after activation of the preserved strain on the slant surface of the PDA medium in the incubator at 25 °C, four mycelium blocks (5 × 5 mm) were removed and placed in 500 mL Erlenmeyer flasks containing 150 mL of liquid medium. The samples were incubated for four days on a shaker (140 r min^−1^) at 22 °C.

The culture flasks were loaded with fruiting-body culture medium and sterilized at 121 °C for 25 min. After cooling, 5 mL of the strain suspension was added to each flask and cultured in the dark for seven days at 22 °C until the mycelia completely covered thef the bottom of the flask. After stimulation of the mycelia, the culture was incubated at 18 °C with 80–90% humidity and 8% CO_2_ under continuous light (200lx) for 45 days. Cultures were harvested after maturation of the ascospores.

Identification of mating-type genes: Genomic DNA was extracted from the strains. Based on the sequences of the mating genes MAT 1-1 (AB194982.1) and MAT 1-2 (AB084257.1) in the NCBI database, Primer 5.0 was used to design specific primers with sequences from 5′ to 3′ as follows: MAT1-1 F (GAGCCTACTATGGAACCC), MAT1-1 R(CAGGACTGATACCAGCAAA) and the MAT1-2 F(GCATCAACCCATTTGTGAAAGTTCT), MAT1-2R(CCTGTCATAATGGTGCTGT). The amplified fragment of MAT1-1 was 427 bp and the amplified fragment of MAT1-2 was 719 bp. The PCR parameters were: 94 °C for 3 min; 30 cycles of 94 °C for 30 s, 56 °C for 30 s, 72 °C for 30 s, and a final extension of 72 °C for 10 min. The PCR products were analyzed by electrophoresis on 1% agarose gels. The primer pair sequences and amplification procedures were as previously described (Feng, 2019).

#### Isolation, identification, and proportion determination of single-conidium strains

Isolation of single-conidium strains: Gradient dilution isolation of conidia was used to isolate single-conidium strains. Under aseptic conditions, 0.5 × 0.5 cm blocks of mycelial culture were placed in Erlenmeyer flasks containing 50 mL of sterile water and shaken for 1 min before filtration with sterilized two-layer lens paper to recover the conidial suspension. The filtrate was serially diluted with sterile water to 10^−1^, 10^−2^, 10^−3^, 10^−4^, and 10^−5^, and 100 µL aliquots of the diluents were spread on PDA medium and cultured in the dark at 25 °C for two to three days. When macroscopic colonies were apparent on the medium surface, a total of 48 single colonies were selected and inoculated on fresh medium with an inoculating needle. The cultures were incubated until the plates were covered with mycelia, and the media were stored at 4 °C for later use.

Molecular identification and verification of single mating-type strains: Single colonies of mycelia were harvested, and the genomic DNA was extracted using a DNA extraction kit (CWBio, Taizhou, China). Mating-type genes were identified using the method described in “Verification of fruiting was performed on the single mating-type strains”.

#### Strain subculture and fruiting test

Single (MAT1-1 and MAT1-2) and double mating-type mycelia in the six strains were subcultured every 15 days by selecting flat plates with even mycelial growth from the previous generation. The nascent mycelia at the edges of the plates were removed with an inoculating needle, inoculated on fresh PDA medium, and incubated at 25 °C for 15 days before use.

**Figure 1:**
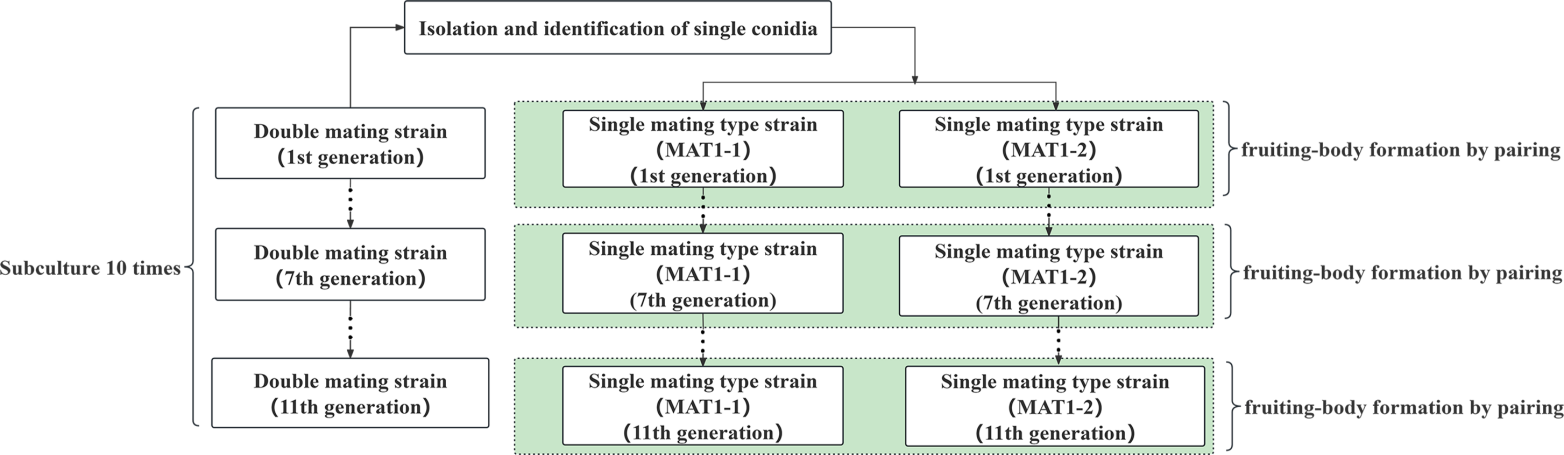
Diagram of single and double mating hypha subculture and fruiting experiment.

First- to eleventh-generation strains were obtained through the transfer and subculture method, and the experimental processes of subculture and fruiting are shown in Fig. 1. After completion of the subculture, 1 × one cm blocks were collected from the double mating-type strain for liquid culture, while the same generations of the MAT1-1-type and MAT1-2-type strains were collected from the single mating-type strain for liquid culture. Fruiting analysis was conducted by mixing equal volumes of the strains. Fruiting experiments were conducted on the first-, third-, fifth-, ninth-, and eleventh-generation strains using 5 mL volumes of inoculum in fruiting-body culture medium. Fruiting bodies were cultured as described in “Verification of fruiting was performed on the single mating-type strains”, and once the fruiting body was mature, it was harvested and dried to constant weight for weight measurement.

#### Determination of the proportion of conidia in the different mating types in degenerated strains

Single conidia were isolated from the degenerated strains using the gradient dilution method described in “Isolation, identification, and proportion determination of single-conidium strains”. Forty-eight single-conidia strains were randomly selected and their mating types were identified, as described in “Isolation, identification, and proportion determination of single-conidium strains”, to determine the proportions of the two mating types.

#### Effect of inoculation with different proportions of the two mating type strains on production

Measurement of mycelium concentration: Mycelium concentrations of the two mating-type liquid cultures were determined using the wet weight measurement method. Different single mating-type strains were cultured in liquid, and 10 mL aliquots of each fermentation broth were centrifuged. After discarding the supernatant, the mycelia were weighed to determine their concentrations in the two liquid spawns.

Determination of fruiting of strains inoculated with different proportions of the two mating types: MAT1-1 and MAT1-2-type strains in proportions of 1: 9, 2: 8, 3: 7, 4: 6, 5: 5, 6: 4, 7: 3, 8: 2, and 9: 1 were constructed according to the mycelium concentrations, using 5 mL volumes. The mixtures were then inoculated on fruiting medium for fruiting analysis. Fruiting bodies were cultured as described in “Verification of fruiting was performed on the single mating-type strains” and harvested when mature, after which the fruiting body was dried to a constant weight for measurement.

#### Data processing and statistical analysis

Data processing was performed using SPSS 20.0, using Duncan’s multiple range test to determine data significance. Graphs were plotted using GraphPad Prism 8. A statistical difference was considered when *P*<0.05. Chi-square tests were performed as previously described (Lin et al., 2000).

## Results

### Fruiting verification and mating-type gene identification of strains

**Figure 2:**
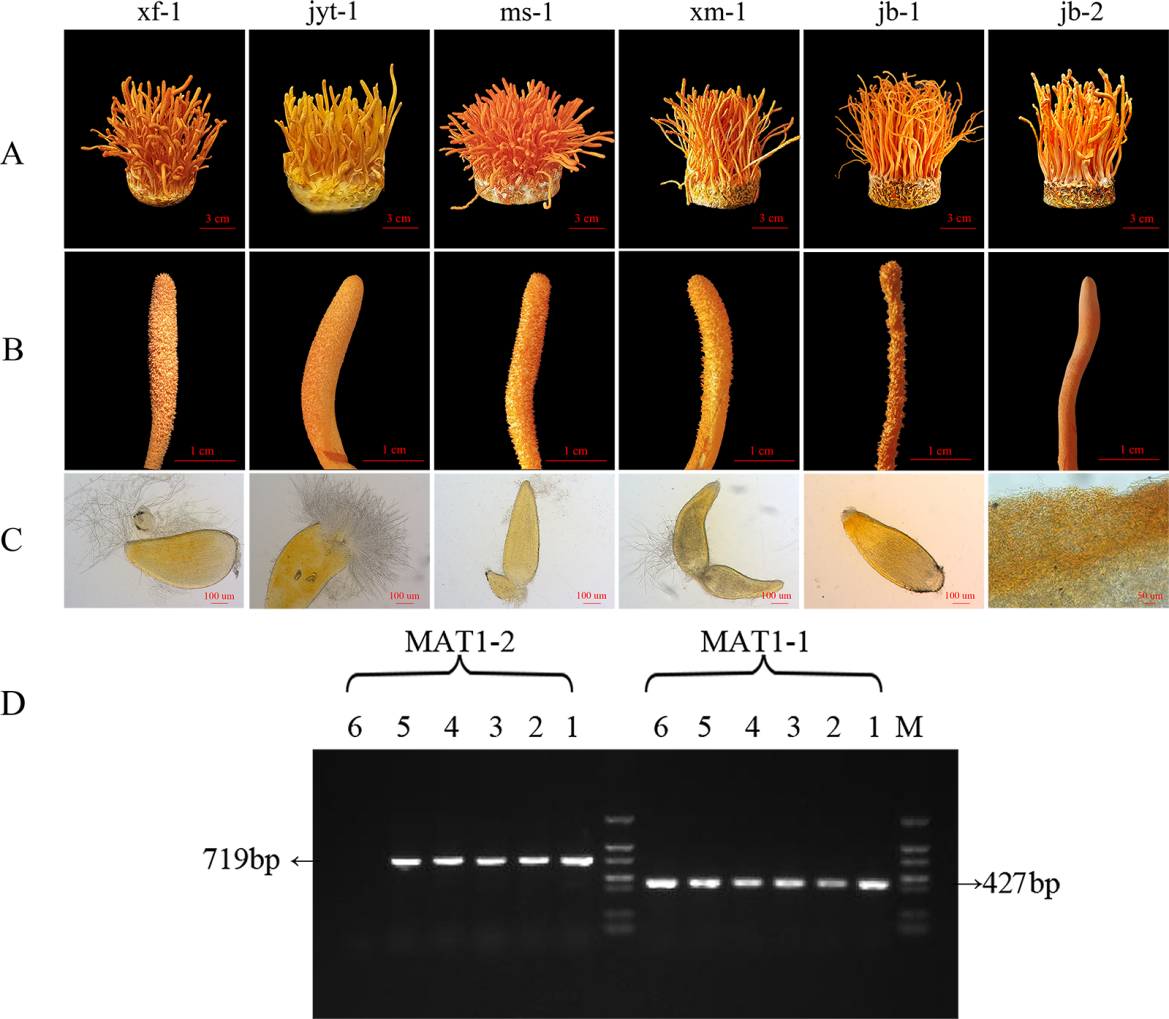
Morphology and mating type gene identification of the original strains in *Cordyceps militaris*. (A) Morphology of the fruiting bodies of the six strains (B) Enlarged view of the morphology of the six strains (C) Microstructure of the perithecia of the six strains; strain jb-2 had no perithecia. (D) Identification of MAT genotypes of the test strains by PCR amplification. 1: xf-1 strain; 2: jyt-1 strain; 3: ms-1 strain; 4: xm-1 strain; 5: jb-1 strain; 6: jb-2 strain.

Effective fruiting was observed in all six strains (Fig. 2A). All six strains showed a relatively high biological efficiency, while the production strain jb-1 had a biological efficiency of over 140% (Table 1).

**Table 1:** The fruiting biological transformation efficiency and dry weights of the six strains.

Strain	Biological efficiency (%)	Dry weight (g)
xf-1	123.43 ± 7.00	6.96 ± 0.49
jyt-1	119.43 ± 5.98	6.93 ± 1.26
ms-1	136.44 ±13.87	7.5 ± 0.29
xm-1	123.07 ± 9.74	7.14 ± 0.21
jb-1	141.94 ± 14.68	8.53 ± 0.3
jb-2	135.36 ± 3.38	8.44 ± 0.72

**Notes.**

Values are expressed as the mean ± standard deviation.

After analysis of the ascospores traits and identification of the mating-type genes in the strains, it was found that five strains grew normal perithecia and could produce ascospores (Figs. 2A, 2B and 2C). These five strains had both MAT1-1 and MAT1-2 mating-type genes and could thus be classified as double mating-type strains (Fig. 2D). The surface of the fruiting body in the jb-2 strain appeared smooth, without perithecia or ascospores (Fig. 2C), and only the MAT1-1 gene was identified in this strain (Fig. 2D). The jb-2 strain thus represented a mutant single mating-type strain that was able to fruit constantly but did not produce ascospores.

### Isolation, identification, and proportion determination of single-conidium strains

**Figure 3:**
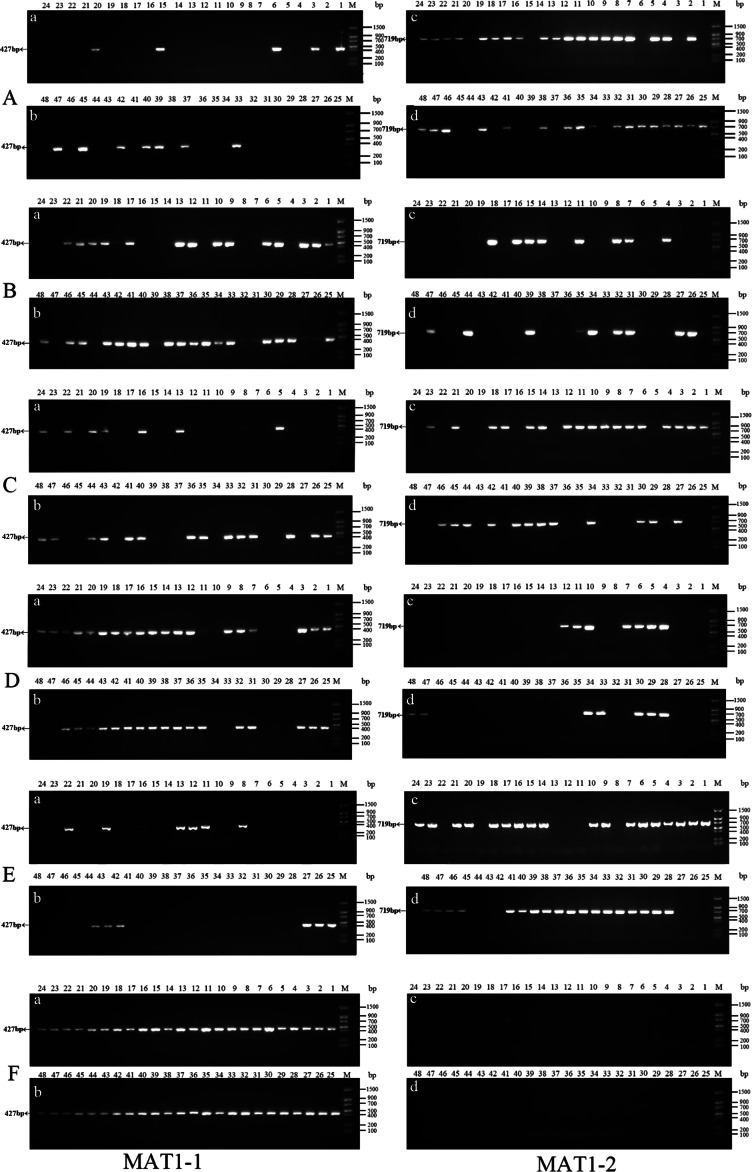
Identification of MAT genotypes of single-conidium isolated from original strains by PCR amplification. (A) xf-1 strain; (B) jyt-1 strain; (C) ms-5 strain; (D) xm-1 strain; (E) jb-1 strain; (F) jb-2 strain.

Forty-eight single conidia were randomly selected from the first generation of each strain to determine the mating-type ratio. The results showed that the proportions of the MAT1-1 and MAT1-2 type single conidia differed between the different strains, and the chi-square test showed that only strain ms-1 had a 1:1 ratio (*P*<0.05). The dominant mating type of the xf-1 and jb-1 strains was MAT1-2, while the MAT-1 type was observed in the xm-1 and jyt-1 strains. Only one mating type (MAT-1-1) was observed in strain jb-2. The remaining strains showed two mating types, with a probability of 2.1 − 4.2%. It has not yet been determined whether this was because the conidia of the different mating types did not separate or whether it resulted from a heterokaryon single conidium (Table 2, Fig. 3).

### Strain subculture and fruiting tests

**Figure 4:**
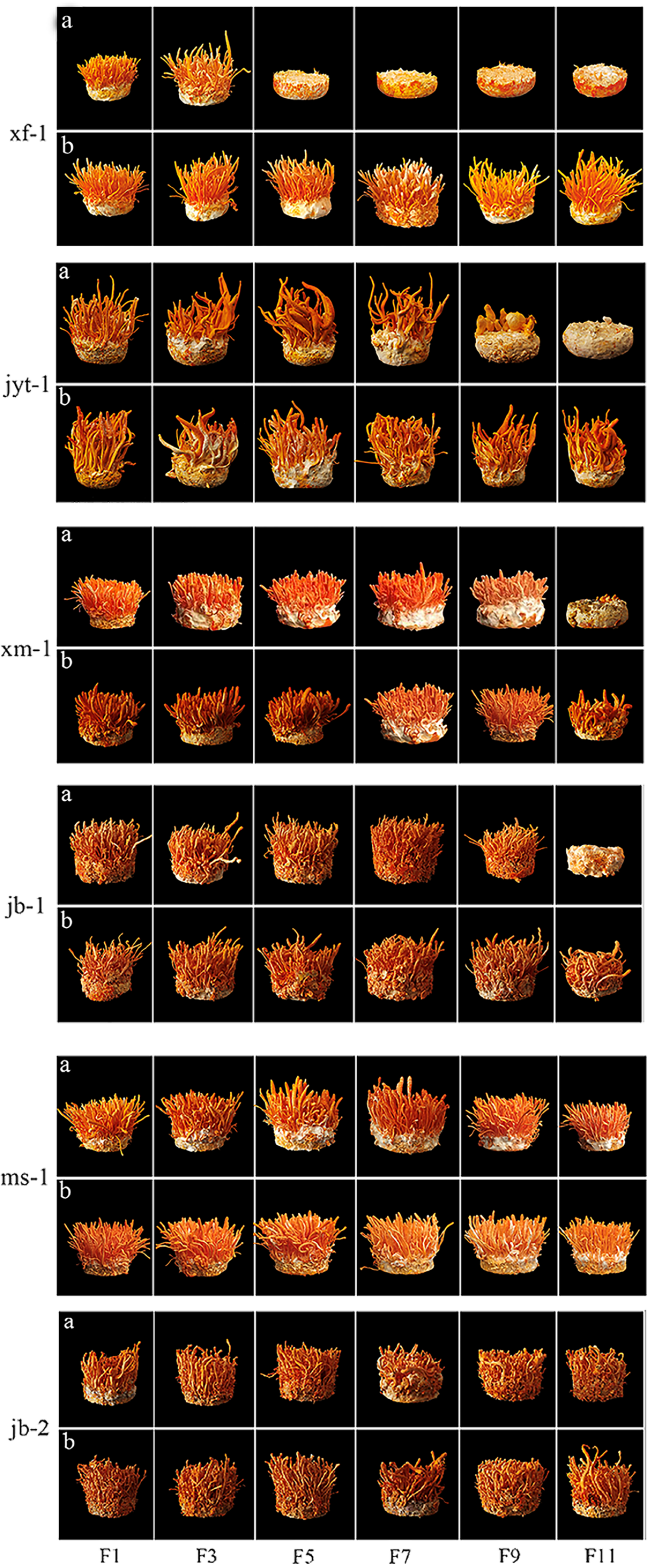
Fruiting body formation of double mating strain were subcultured and the single conidia strains were subcultured for paired fruiting. (A) Fruiting body formation of double mating strain were subcultured. (B) The single conidia strains were subcultured for paired fruiting.

Changes in two agronomic traits, namely, a gradual reduction in yield and a complete degeneration without growth, can occur during subculture (Tables 3 and 4, and Fig. 4). An inability to fruit occurs in most double mating-type strains during subculture. The wild strains xf-1 and jyt-1 were found to have degenerated completely by the 5th and 11th generations, respectively, while the production strains xm-1 and jb-1 showed complete degeneration by the 11th generation. Subculturing single mating-type mycelia separately and mixing them before production effectively mitigated degeneration during subculture.

**Table 2:** Mating types and proportions in single-conidium strains.

strain	Mating type and proportion of single-conidium strain			
	*MAT1-1*	*MAT1-2*	*MAT1-1* and *MAT1-2*	*MAT1-1*/MAT1-2^*^
xf-1	12	35	1	1: 3
jyt-1	31	15	2	2: 1
ms-1	19	27	2	1: 1
xm-1	34	12	2	3: 1
jb-1	12	36	0	1: 3
jb-2	48	0	0	1: 0

**Notes.**

* *P* < 0.05 by chi-square test.

**Table 3:** Dry weight of the fruiting body of *Cordyceps militaris* by the subculture of double mating-type mycelia.

Strain	Generations					
	1	3	5	7	9	11
xf-1	4.87 ± 0.47a	2.86 ± 0.78b	–	–	–	–
jyt-1	4.45 ± 1.13a	3.55 ± 0.75bc	5.54 ± 0.47a	3.76 ± 0.84bc	2.35 ± 0.35c	–
ms-1	6.53 ± 0.46a	6.67 ± 0.25a	6.56 ± 0.30a	5.73 ± 0.43ab	5.26 ± 0.63b	6.1 ± 0.46ab
xm-1	2.93 ± 0.15bc	4.75 ± 0.65a	4.45 ± 0.80a	3.79 ± 0.45ab	2.1 ± 0.21c	–
jb-1	8.23 ± 0.21a	8.53 ± 0.24a	8.36 ± 0.32a	3.92 ± 0.92b	2.29 ± 0.23c	–
Jb-2	6.53 ± 0.09c	7.53 ± 0.64abc	8.47 ± 0.92ab	6.76 ± 0.84bc	9.07 ± 0.10a	7.74 ± 1.16abc

**Notes.**

Data were analyzed with Duncan’s multiple range test. Mean values within a column followed by the same letter do not differ significantly (*P* > 0.05).

**Table 4:** Dry weight of the fruiting body of *Cordyceps militaris* by the subculture of single mating-type mycelia.

Strain	Generations					
	1	3	5	7	9	11
xf-1	3.85 ± 0.60b	4.46 ± 0.40ab	3.29 ± 0.76b	4.47 ± 0.44ab	4.49 ± 0.92ab	4.59 ± 0.39ab
jyt-1	6.57 ± 1.13a	4.90 ± 1.26a	5.07 ± 0.41a	6.58 ± 0.43a	5.78 ± 0.35a	5.15 ± 0.66a
ms-1	5.36 ± 0.19a	4.03 ± 0.12d	5.90 ± 0.51a	4.67 ± 0.39bcd	4.51 ± 0.24cd	5.06 ± 0.47bc
xm-1	5.73 ± 0.64ab	5.66 ± 0.35ab	5.95 ± 0.28a	4.97 ± 0.25bc	4.1 ± 0.14d	4.24 ± 0.17cd
jb-1	7.80 ± 0.63a	6.21 ± 0.46b	6.63 ± 0.64b	6 ± 0.23b	5.96 ± 0.24b	2.7 ± 0.29c
Jb-2	10.46 ± 0.90a	10.55 ± 0.65a	9.45 ± 0.86ab	9.06 ± 0.52ab	8.64 ± 0.33b	9.44 ± 0.79ab

**Notes.**

Data were analyzed with Duncan’s multiple range test. Mean values within a column followed by the same letter do not differ significantly (*P* > 0.05).

The decrease of fruiting body yield was different in the two ways of double mating type strain subculture and single conidium strain subculture respectively. Single-conidial strains were subcultured to the 11th generation, observing that reductions in the yields of the two producing strains occurred in the 3rd (jb-1) and 9th (xm-1) generations. Fruiting bodies production by the wild strains xf-1 and jyt-1 was still stable in the 11th generation, and there were no obvious decreases in fruiting body yield. Double mating-type strains were subcultured to the 11th generation, with the wild strain (xf-1) starting to decline in yield as early as the 3rd generation and the jyt-1 strain starting to decline in yield by the 7th generation. Reduced yields were apparent in the two production strains (jb-1 and xm-1) in the 7th and 9th generations.

The wild ms-1 (double mating type) strain was exceptional as it continued to stably develop fruiting bodies in the 11th generation and showed significant differentiation from the other four double mating-type strains. The 11th generation of jb-2 (single mating-type strain) also remained stable without decline, showing good successional stability.

### Isolation of single conidia and identification of mating type in double mating-type strains showing degeneration

**Figure 5:**
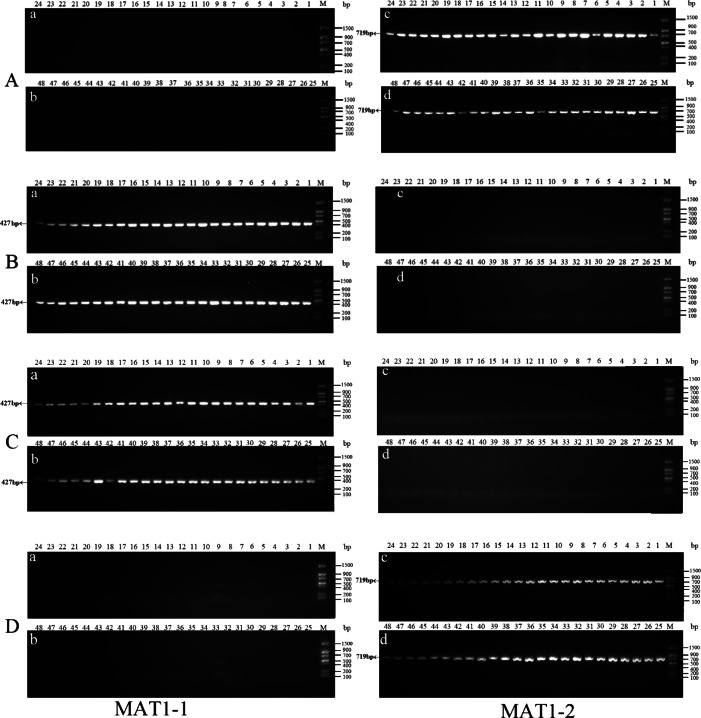
Identification of MAT genotypes of single-conidium in degenerate strains by PCR amplification. (A) xf-1 strain; (B) jyt-1 strain; (C) xm-1 strain; (D) jb-1 strain.

Single conidia were isolated from the degenerated strains with no fruiting bodies, and genes associated with the mating type were identified. It was found that single conidia isolated from four degenerate strains contained only one mating-type gene while the other gene had been completely lost (Table 5; Fig. 5).

**Table 5:** Mating types of single conidia in degenerated strains.

Strain	Mating types of single conidia	
	*MAT1-1*	*MAT1-2*
xf-1	0	48
jyt-1	48	0
xm-1	48	0
jb-1	0	48

Strains xf-1 and jb-1, in which the MAT1-2 type dominated, lacked MAT1-1 type mycelia. Conversely, strains jyt-1 and xm-1, in which the MAT1-1 type dominated, lacked MAT1-2 type mycelia. These findings indicate that the loss of a specific mating-type mycelium (MAT1-1 or MAT1-2) during strain subculture occurs randomly, without predisposition.

### Effects of combining two mating-type strains with different proportions on fruiting

**Figure 6:**
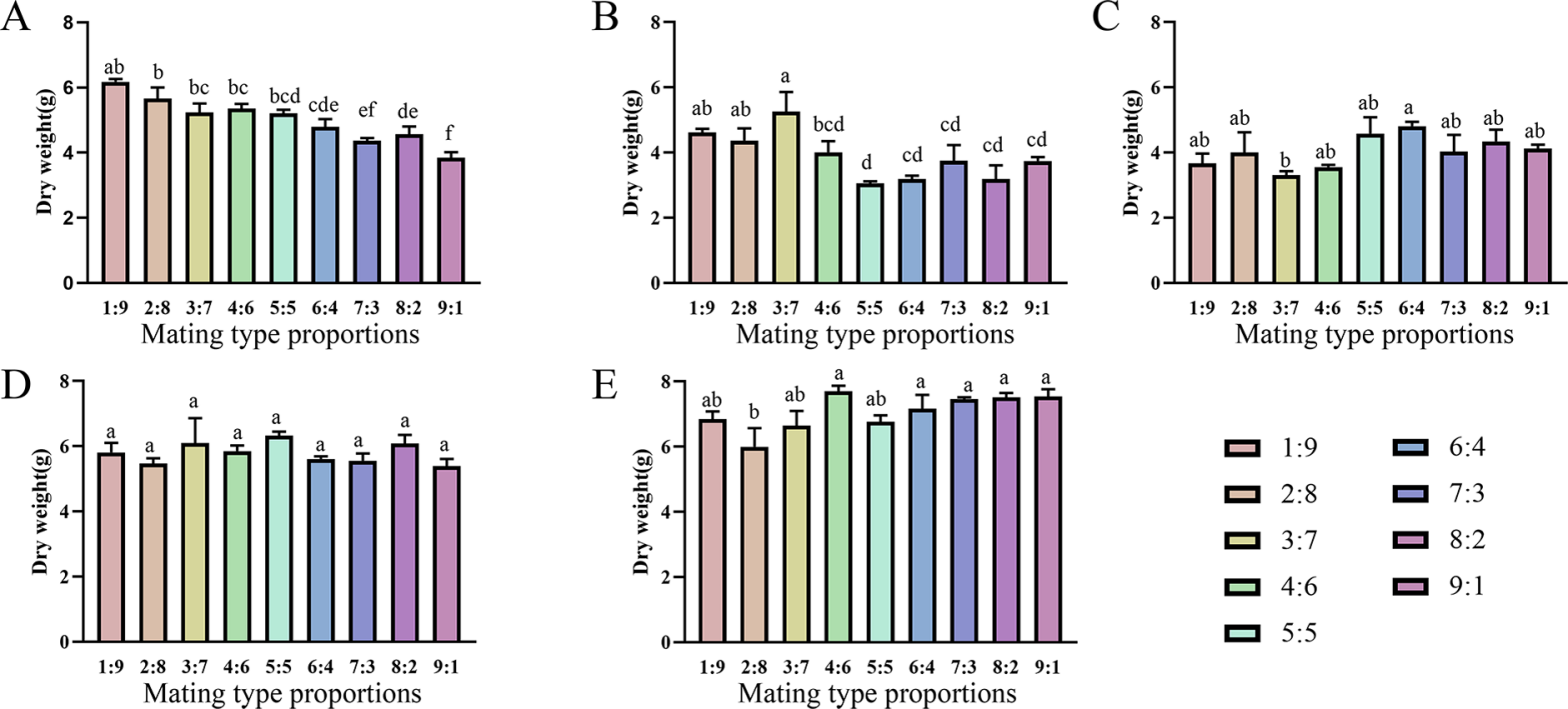
Comparison of fruiting body yield of strains with different proportions of mating type. (A) xf-1 strain. (B) jyt-1 strain. (C) ms-1 strain. (D) xm-1 strain. (E) jb-1 strain. Experiments were conducted in triplicate, and data are presented as means ± standard error. Error bars represent the standard error, and different lowercase letters indicate significant differences (*p* < 0.05).

**Figure 7:**
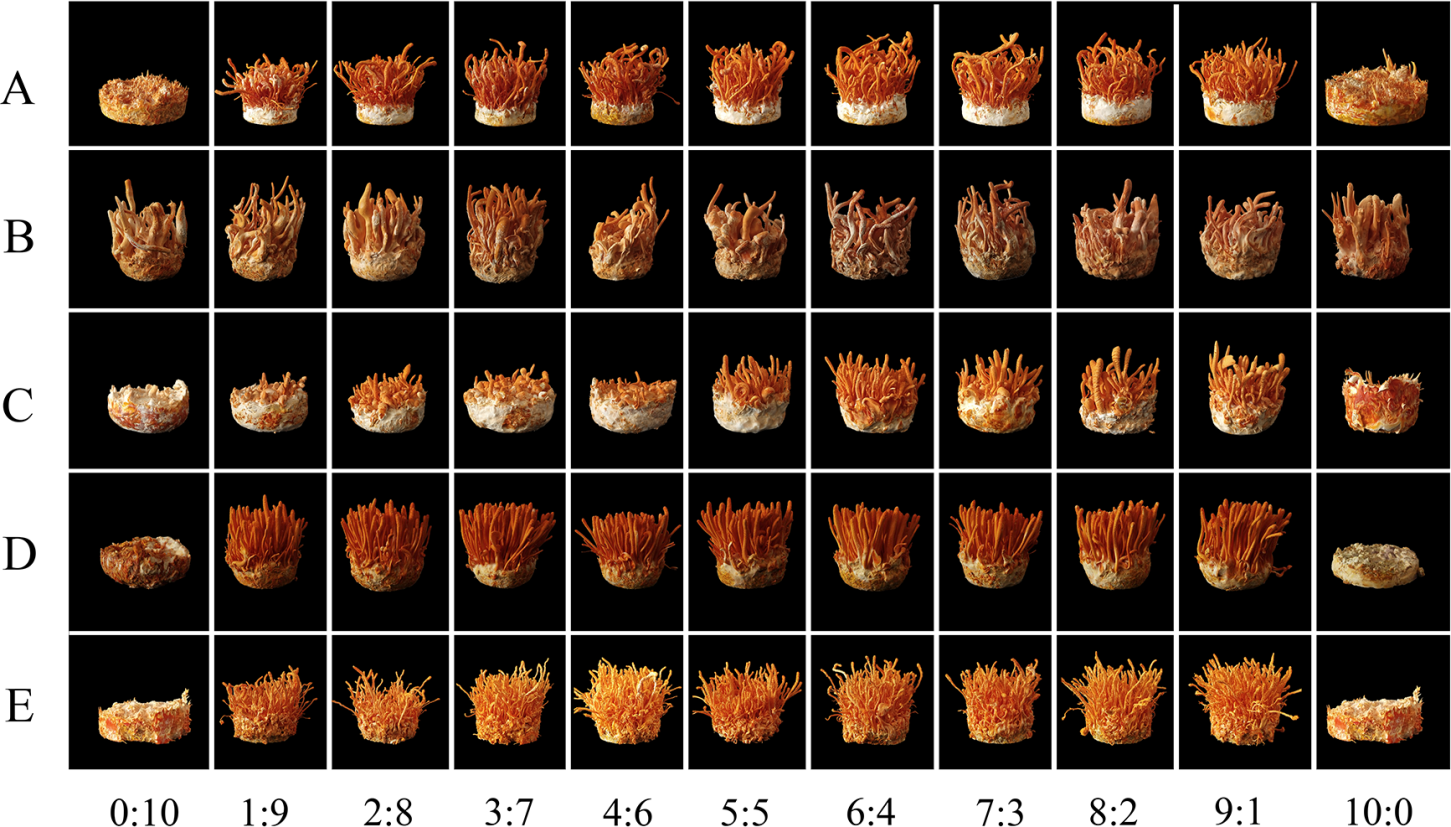
Fruiting body morphology of strains with different proportions of mating type. (A) xf-1 strain. (B) jyt-1 strain. (C) ms-1 strain. (D) xm-1 strain. (E) jb-1 strain.

Varying proportions of the two mating-type strains were inoculated into fruiting medium to evaluate their fruiting abilities (Figs. 6 and 7). The two production strains, xm-1, and jb-1, did not differ significantly in terms of fruiting-body dry weight regardless of the matin-type proportion, indicating that the proportions of the mating-type genes MAT1-1 and MAT1-2 did not influence the development of the fruiting body. Therefore, given the breeding history of these production strains, the proportions of mating-type genes did not significantly affect yield, favoring production stability.

Various mating-type strain combinations had different effects on the formation offruiting bodies in three wild strains. Fruiting-body production declined significantly in strain xf-1 (*P*<0.05) as the proportion of MAT1-2 mating-type genes decreased. MAT1-1:MAT1-2 (9:1) ratios led to the lowest fruiting-body dry weights observed, while the yields of jyt-1 and ms-1 were not associated with the proportions of the MAT1-1 and MAT1-2 genes. The varying impact on yields, greater in strains such as xf-1 and lesser in xm-1 and jb-1, could be attributed to deviations in mating type proportions during subculture.

## Discussion

Several hypotheses could explain the rapid degeneration of *Cordyceps militaris* strains during subculture. These include changes in the mating type, genetic mutations, changes in gene methylation levels, alterations in energy metabolism, and the accumulation of harmful substances in the cell (Wang et al., 2010; Li, 2003; Sun et al., 2017a; Sun et al., 2017b; Li et al., 2007; Xiong et al., 2013; Yin et al., 2017a; Yin et al., 2017b; Xin et al., 2019). Among these, changes in mating type emerge as the most plausible explanation. Studies have shown that strains showing degeneration contain only one mating-type gene, while normal strains contain two mating-type genes (Sun et al., 2017a; Sun et al., 2017b; Wang, 2021; Feng, 2019). Wang et al. (2015) found that about two-thirds of conidia, possessed only one mating-type gene, and classified these as homokaryons. These grew faster than heterokaryotic conidia, indicating a shift from heterokaryon to homokaryon during subculture or transfer. In the present study, the numbers of MAT1-1- and MAT1-2-type genes produced by 48 single conidia of the six strains differed stochastically. Although strains with two mating types were detected, these were relatively few, and further karyotypic analysis was not conducted. Furthermore, the fruiting bodies from degenerated double mating-type strains that had completely ceased fruiting were all found to have lost one mating-type gene. This gene loss was stochastic, indiscriminately affecting both MAT1-1 and MAT1-2-type genes. Therefore, the loss of mating-type genes and the imbalance in their proportions are identified as primary factors in the degeneration of *Cordyceps militaris* strains.

Compared to double mating type strains, while single-conidial subcultures showed prolonged degeneration times, the yields gradually declined in correspondence with the number of subcultures. This indicates that factors beyond mating-type disproportion also contribute (Feng, 2019). Analysis of genes over different generations in subculture has shown the presence of increasing numbers of mutations in the mating-type genes as the time in culture increased. Similar mutations have been observed in various types of edible fungi during asexual reproduction, resulting in long-term degeneration (Yin et al., 2017a; Yin et al., 2017b; Li et al., 2018). Environmental factors have also been found to have an effect (Fang et al., 2011; He et al., 2009; Zuo, 2015).

Differences were observed in the ability of different strains to change the proportion of two mating types to form fruiting bodies. There was no significant difference between the production strains (xm-1, jb-1) and the wild strains (jyt-1, ms-1) in terms of a change in the ratio of the two mating types, which was consistent with the results of Sung et al. (2006) and Zheng, Xia & Xiao (2011). In the wild strain xf-1, the higher proportion of MAT1-2-containing hyphae resulted in a higher fruiting-body yield, with ratios of MAT1-1:MAT1-2 between 1:9 and 9:1. Previous studies indicated that MAT1-2 controls sexual reproduction in *Cordyceps militaris*, and contributes to the formation of the fruiting body (Lu et al., 2016; Sun et al., 2018). When the proportion of MAT1-1 mating-type mycelia was large (MAT1-1:MAT1-2 = 9:1), the fruiting-body yield decreased significantly, consistent with the decrease in fruiting-body yield when MAT1-1:MAT1-2 = 10:1 found by Vu et al. (2023) who also showed that an excessive increase in the MAT1-2 proportion led to marked reductions in the fruiting-body yield. Therefore, based on previous studies and the results of this study, it can be concluded that an imbalance between the two mating-type strains is the main reason for degeneration during subculture.

The single mating-type strain jb-2, containing only MAT1-1, was found to be stable during subculture, continuing to fruit up to the 11th generation. In most cases, *Cordyceps militaris* is a heterothallic fungus with a heterokaryotic composition containing both MAT1-1 and MAT1-2, while the fruiting body contains only one mating type and cannot form ascospores. The jb-2 strain was found to contain only MAT1-1 mating-type genes, and the surface of the mature fruiting body appeared smooth without perithecia or ascospores, in agreement with the findings of a previous study (Lu et al., 2016). However, the jb-2 strain could fruit stably with excellent genetic stability and has thus been used in the large-scale production of *Cordyceps militaris*. Therefore, additional research is required to understand the unique genetic characteristics of the jb-2 strain, particularly its superior yield trait compared to other single mating-type strains.

In terms of the subculture stability of the *Cordyceps militaris* strains, four aspects can be considered to improve production and breeding. First, single mating-type hyphae should be subcultured separately and then mixed before production and use, as this could effectively solve the problem of rapid deterioration in fruiting performance during subculture. Second, double mating-type strains with yield traits insensitive to the mating-type proportion should be selected for breeding. Compared with wild strains, altering the proportion of the mating type in the production strains xm-1 and jb-1 did not influence the yield significantly, suggesting that breeding and domestication could effectively improve subculture stability. Third, strains with good subculture stability and reduced likelihood of losing mating type should be selected. The wild strain ms-1, for example, was stable on subculture, maintaining its yield up to the 11th generation. Fourth, single mating-type strains with stable fruiting capability should be selected. Although single mating-type strains cannot produce ascospores, they nevertheless have excellent production features and could thus be used for large-scale production.

## Conclusions

An imbalance or loss of mating-type genes has been identified as one of the primary causes of rapid degeneration in *Cordyceps militaris* strains during subculture. Separate subculture of single mating-type mycelia effectively prevents this rapid decline in yield. In terms of the breeding of *Cordyceps militaris*, strains containing both mating types and insensitive to the proportion of mating-type genes are stable in subculture and not prone to mating-type loss and should thus be selected for breeding. Additionally, single mating-type strains with stable fruiting body production are also viable for selection.

##  Supplemental Information

10.7717/peerj.17648/supp-1 Supplemental Information 1 The original uncropped images of MAT genotypes were identified by PCR amplification

10.7717/peerj.17648/supp-2 Data S1 Raw data The fruiting biological transformation efficiency and dry weights of the six strains, dry weight of the fruiting body of Cordyceps militaris by the subculture of double mating-type mycelia, dry weight of the fruiting body of Cordyceps militaris by the subculture of single mating-type mycelia, and a comparison of fruiting body yield of strains with different proportions of mating type.

## Additional Information and Declarations

### Data Availability

The following information was supplied regarding data availability: The data is available in the Supplementary Files.
