# Supplementary figures and images for: Effects of mating-type ratio imbalance on the degeneration of Cordyceps militaris subculture and preventative measures
Source: PeerJ. 2024 Jul 9;12:e17648. doi: 10.7717/peerj.17648 (PMC11243967; doi:10.7717/peerj.17648)

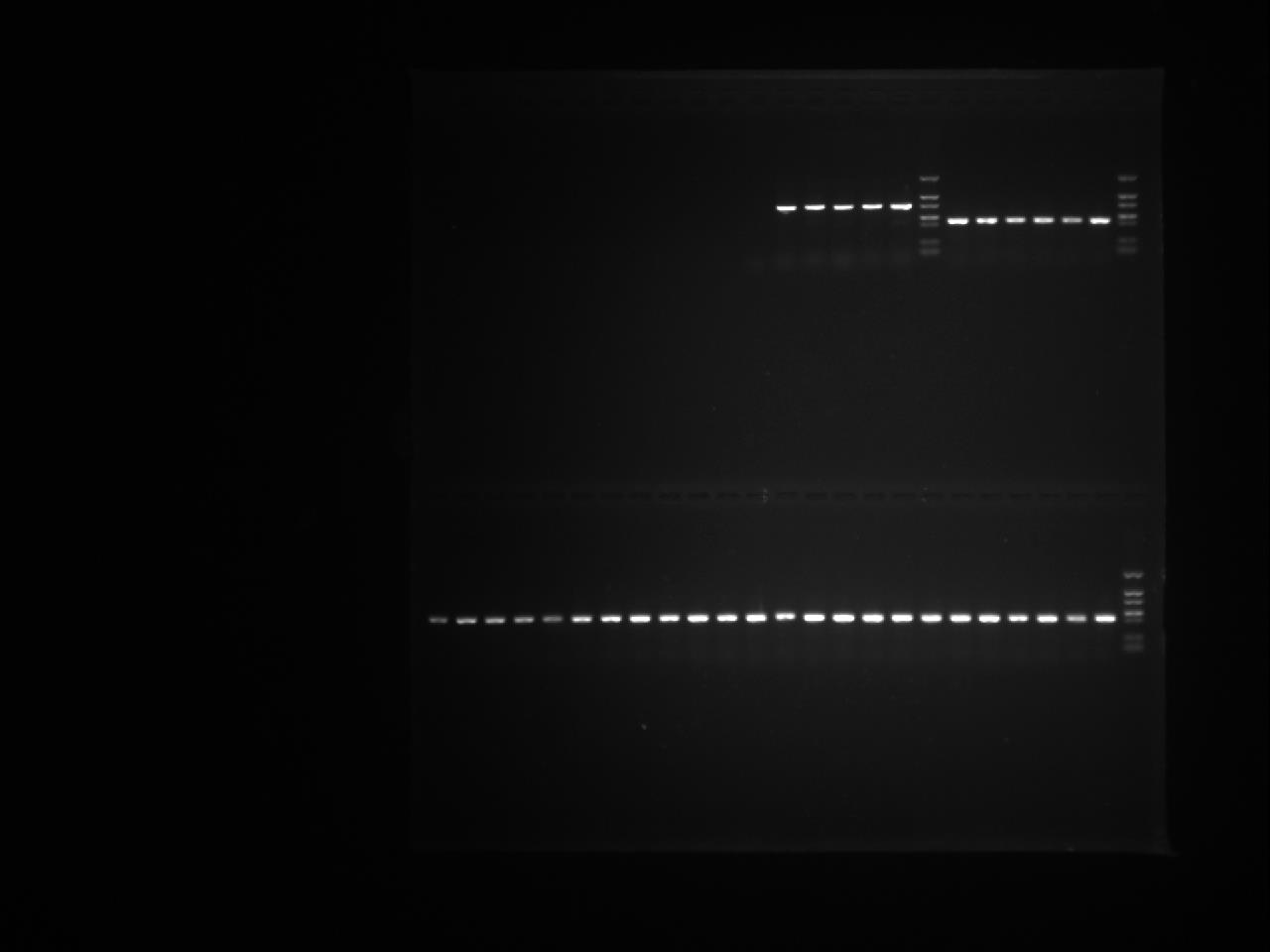

Supplement: Supplemental Information 1 [file peerj-12-17648-s001.zip › original image/Figure 2D.Identification of MAT genotypes of 6 strains tested/Identification of MAT genotypes of 6 strains tested(above).jpg]

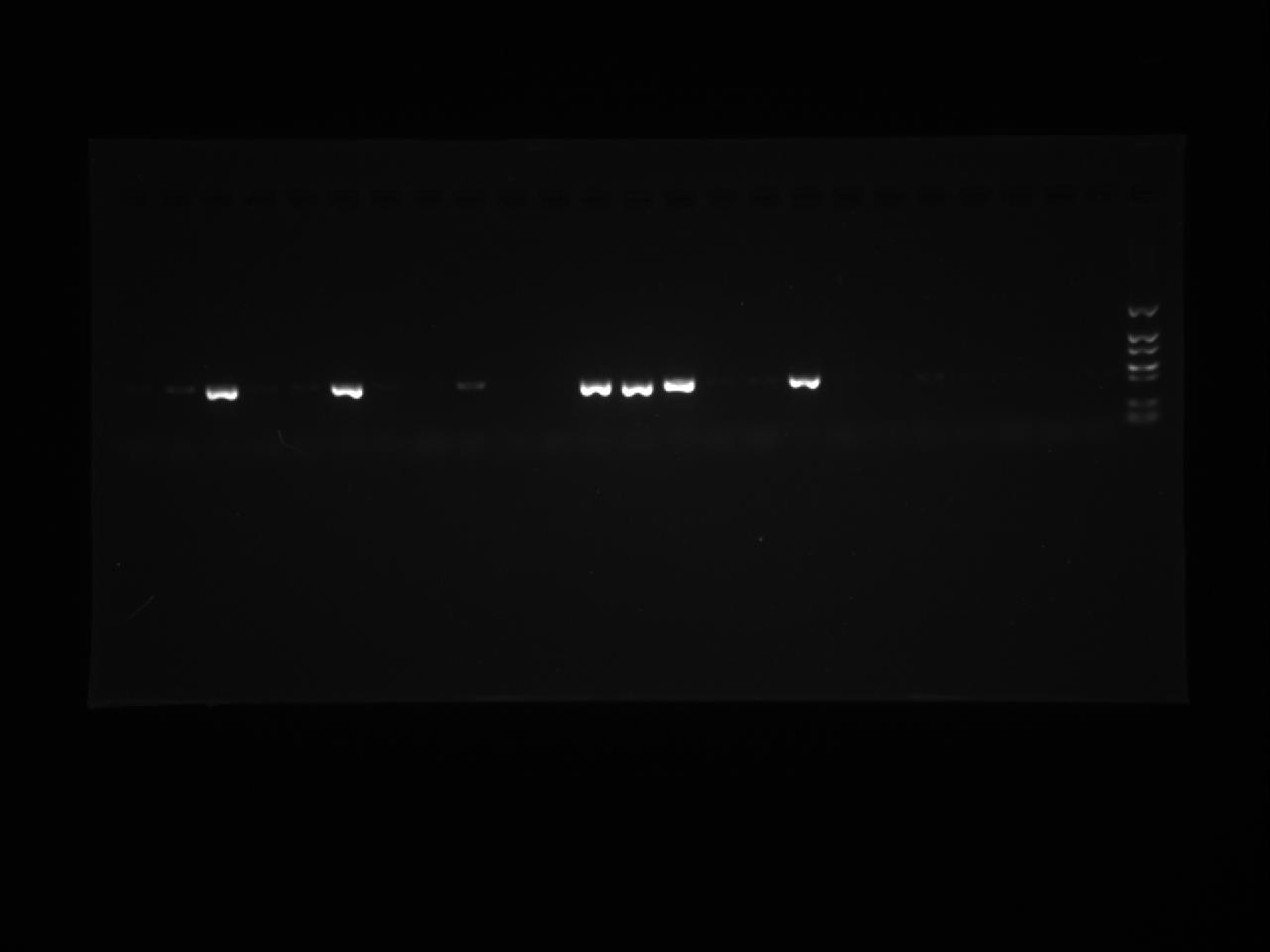

Supplement: Supplemental Information 1 [file peerj-12-17648-s001.zip › original image/Figure 3.Identification of MAT genotypes of single-conidium isolated from F1 strains by PCR amplification/jb-1/jb-1(a).jpg]

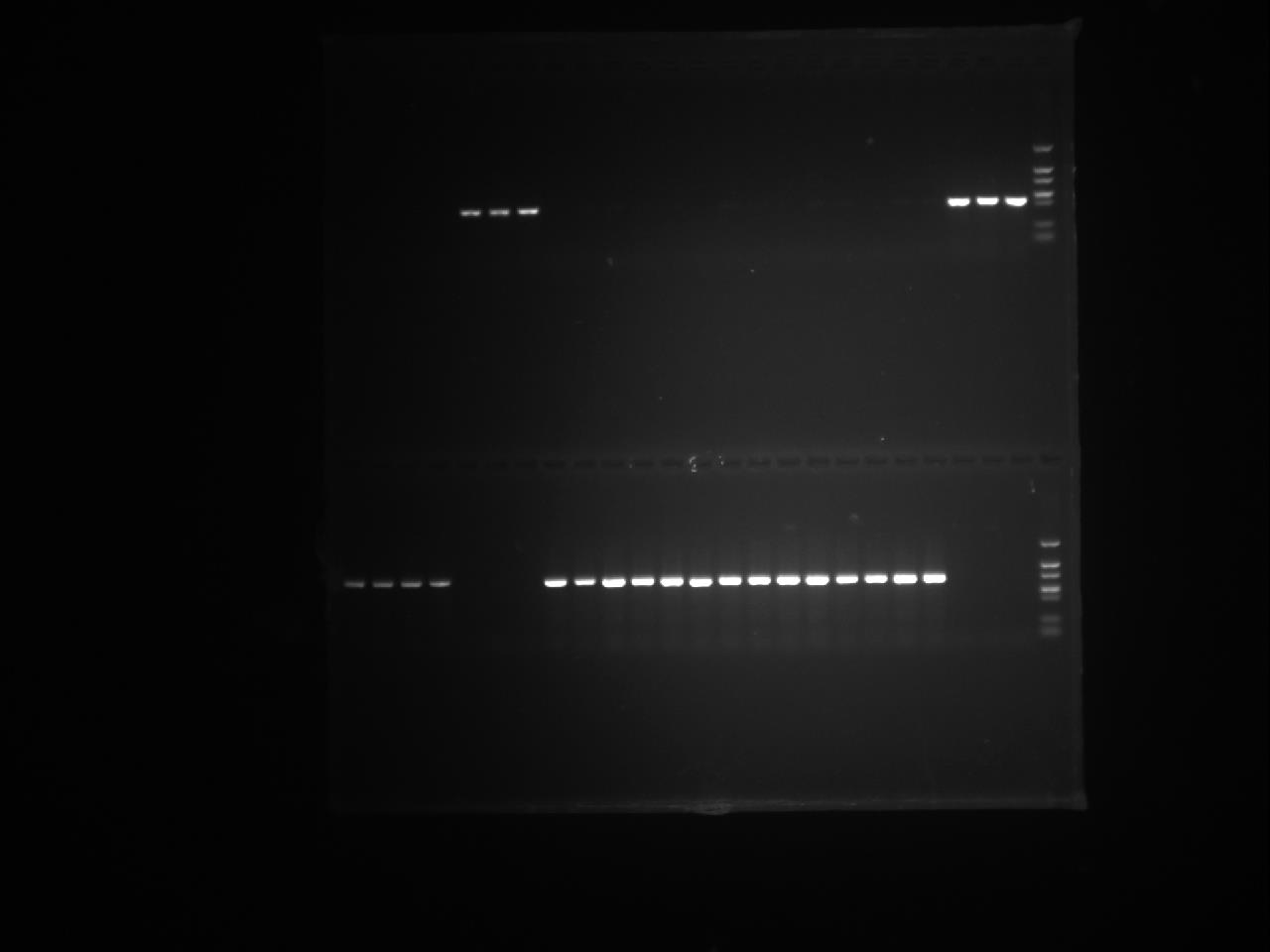

Supplement: Supplemental Information 1 [file peerj-12-17648-s001.zip › original image/Figure 3.Identification of MAT genotypes of single-conidium isolated from F1 strains by PCR amplification/jb-1/jb-1(b,d).jpg]

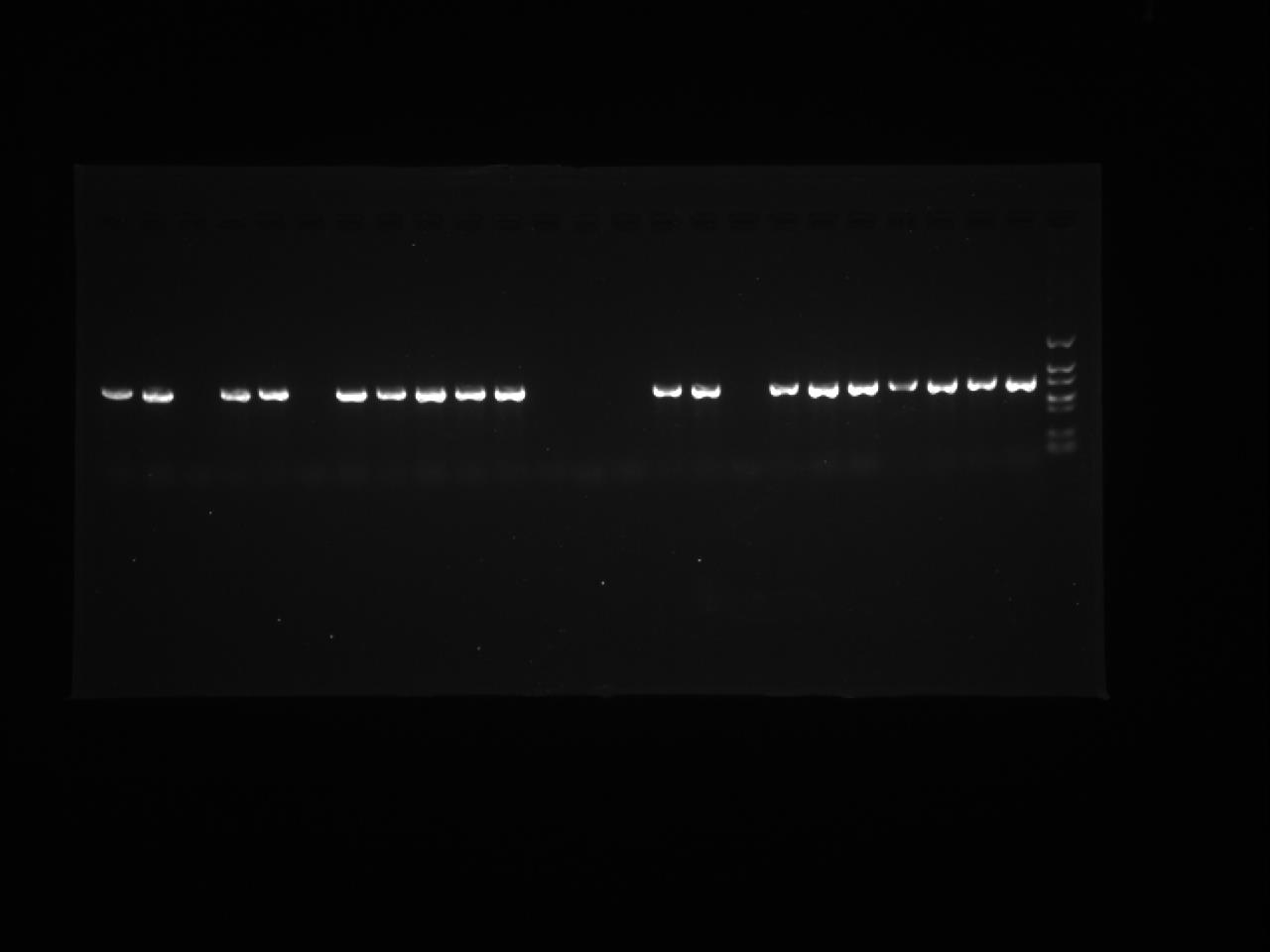

Supplement: Supplemental Information 1 [file peerj-12-17648-s001.zip › original image/Figure 3.Identification of MAT genotypes of single-conidium isolated from F1 strains by PCR amplification/jb-1/jb-1(c).jpg]

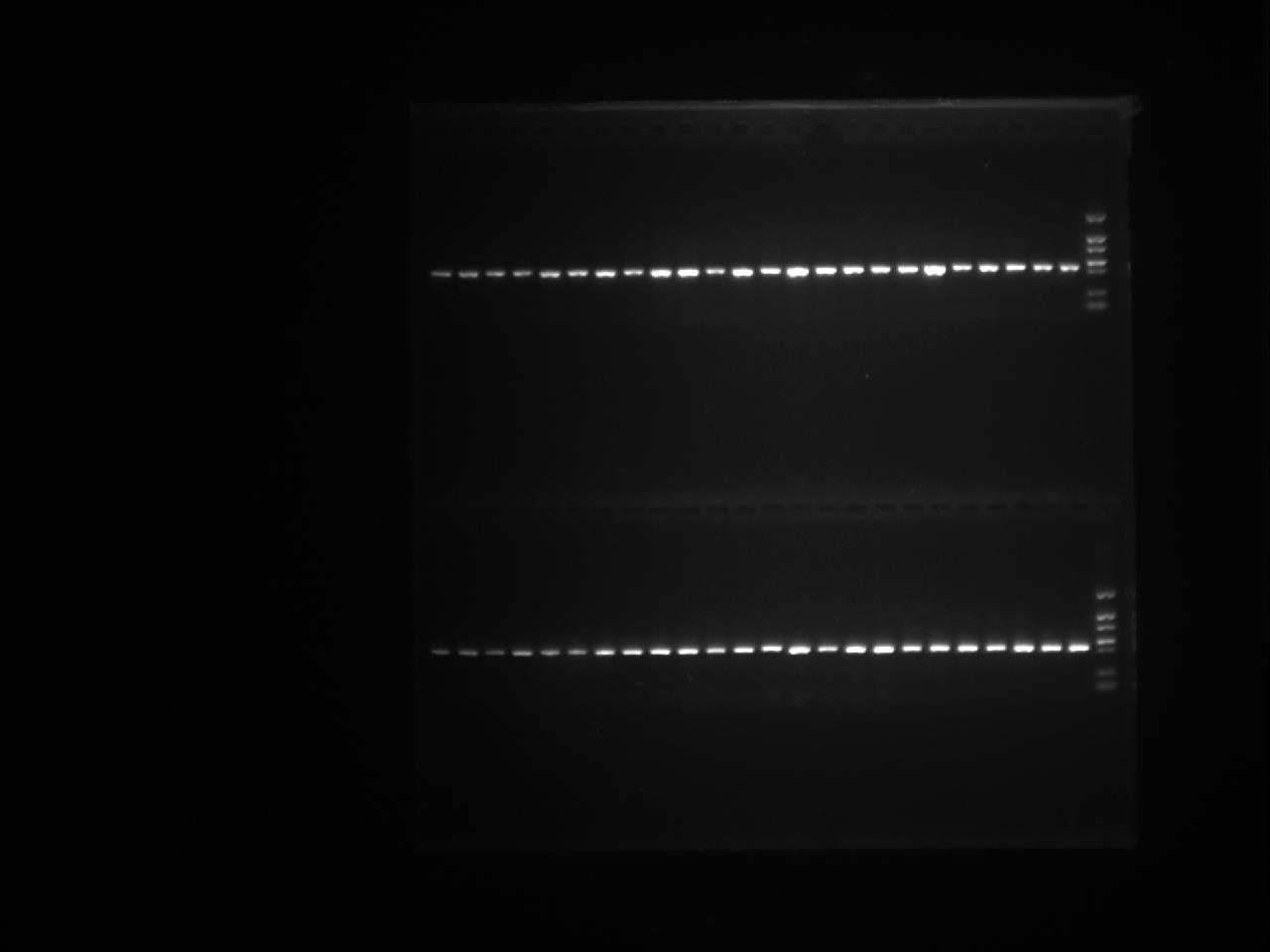

Supplement: Supplemental Information 1 [file peerj-12-17648-s001.zip › original image/Figure 3.Identification of MAT genotypes of single-conidium isolated from F1 strains by PCR amplification/jb-2/jb-2(a,b).jpg]

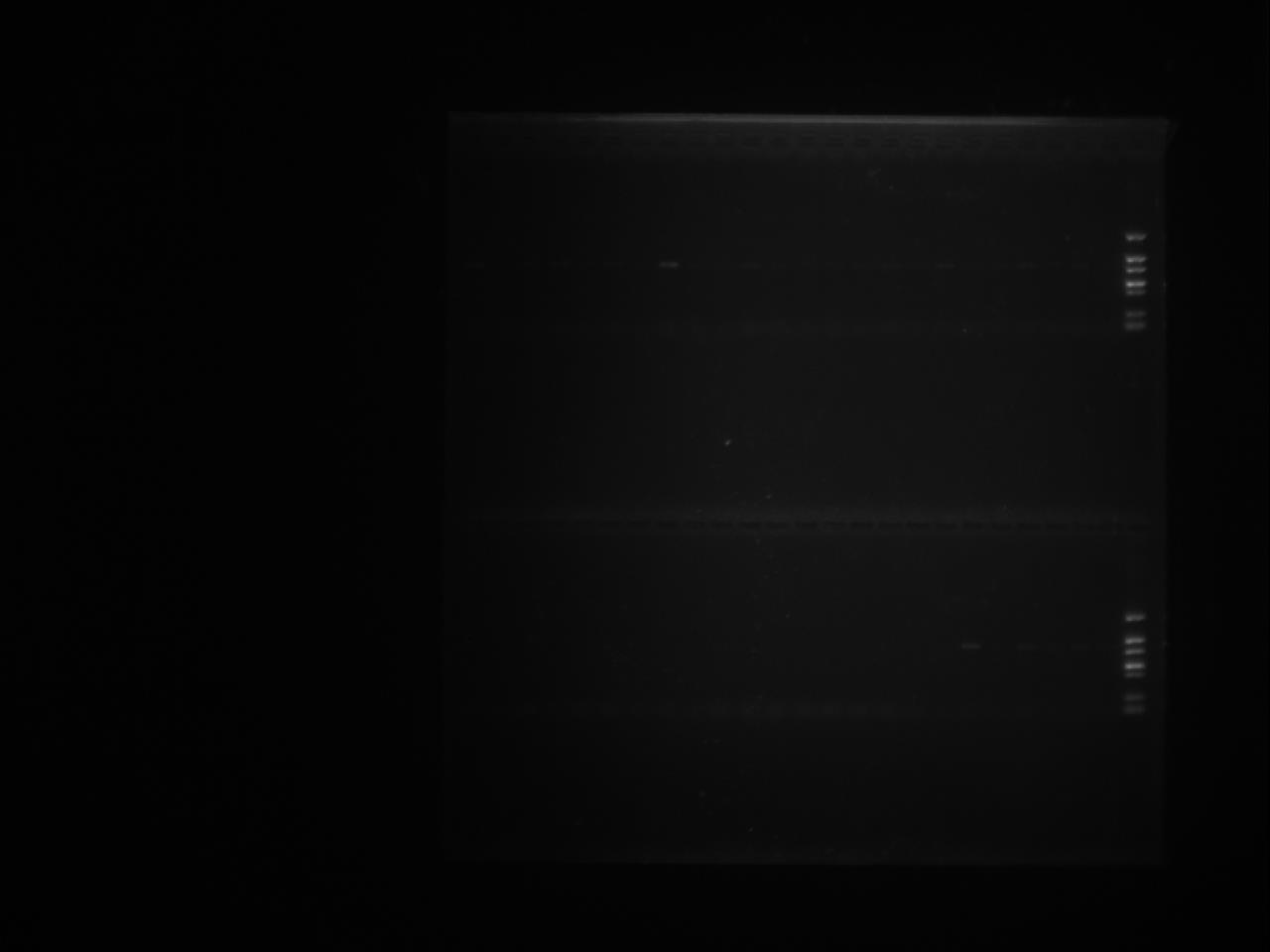

Supplement: Supplemental Information 1 [file peerj-12-17648-s001.zip › original image/Figure 3.Identification of MAT genotypes of single-conidium isolated from F1 strains by PCR amplification/jb-2/jb-2(c,d).jpg]

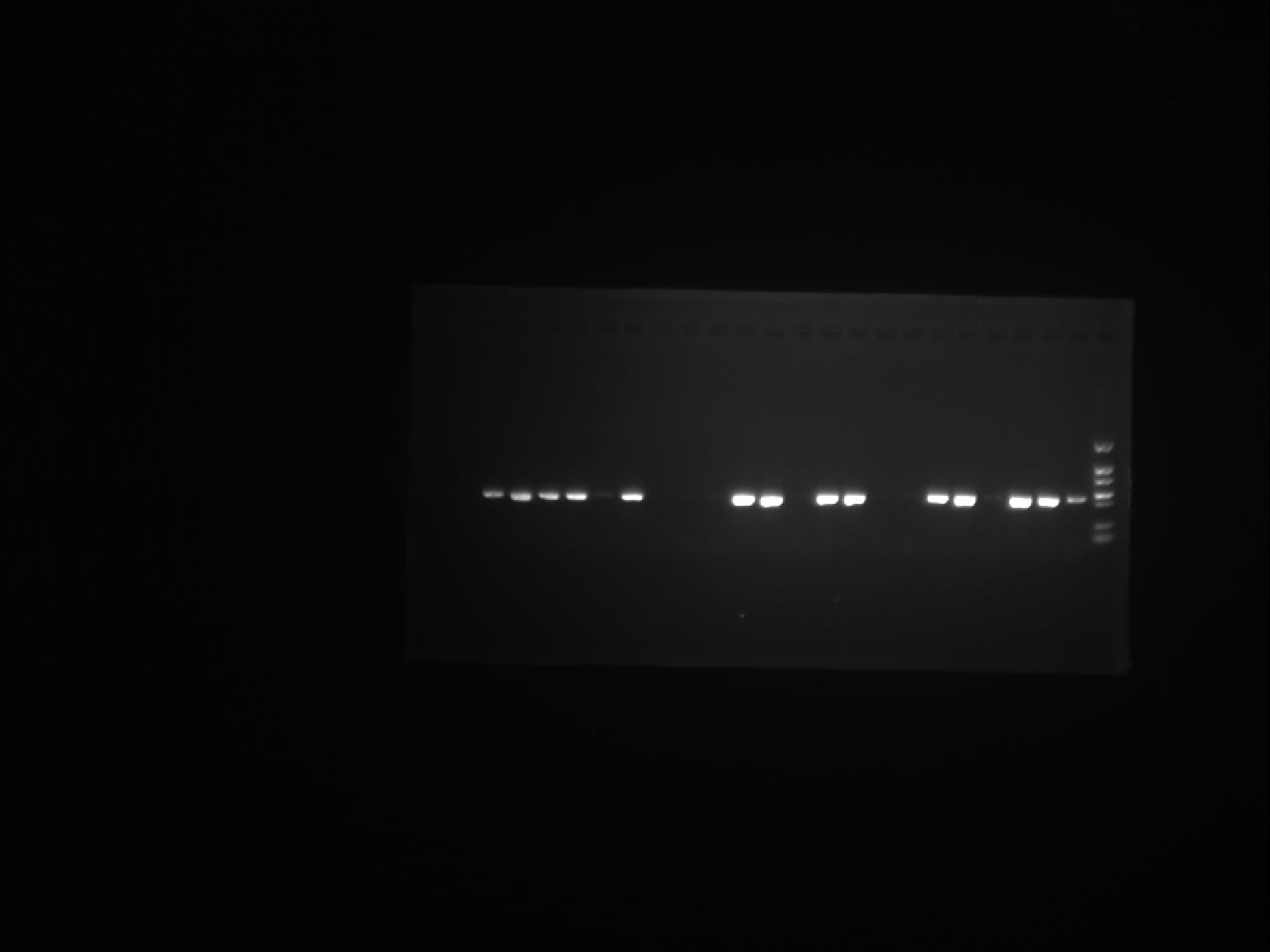

Supplement: Supplemental Information 1 [file peerj-12-17648-s001.zip › original image/Figure 3.Identification of MAT genotypes of single-conidium isolated from F1 strains by PCR amplification/jyt-1/jyt-1(a).jpg]

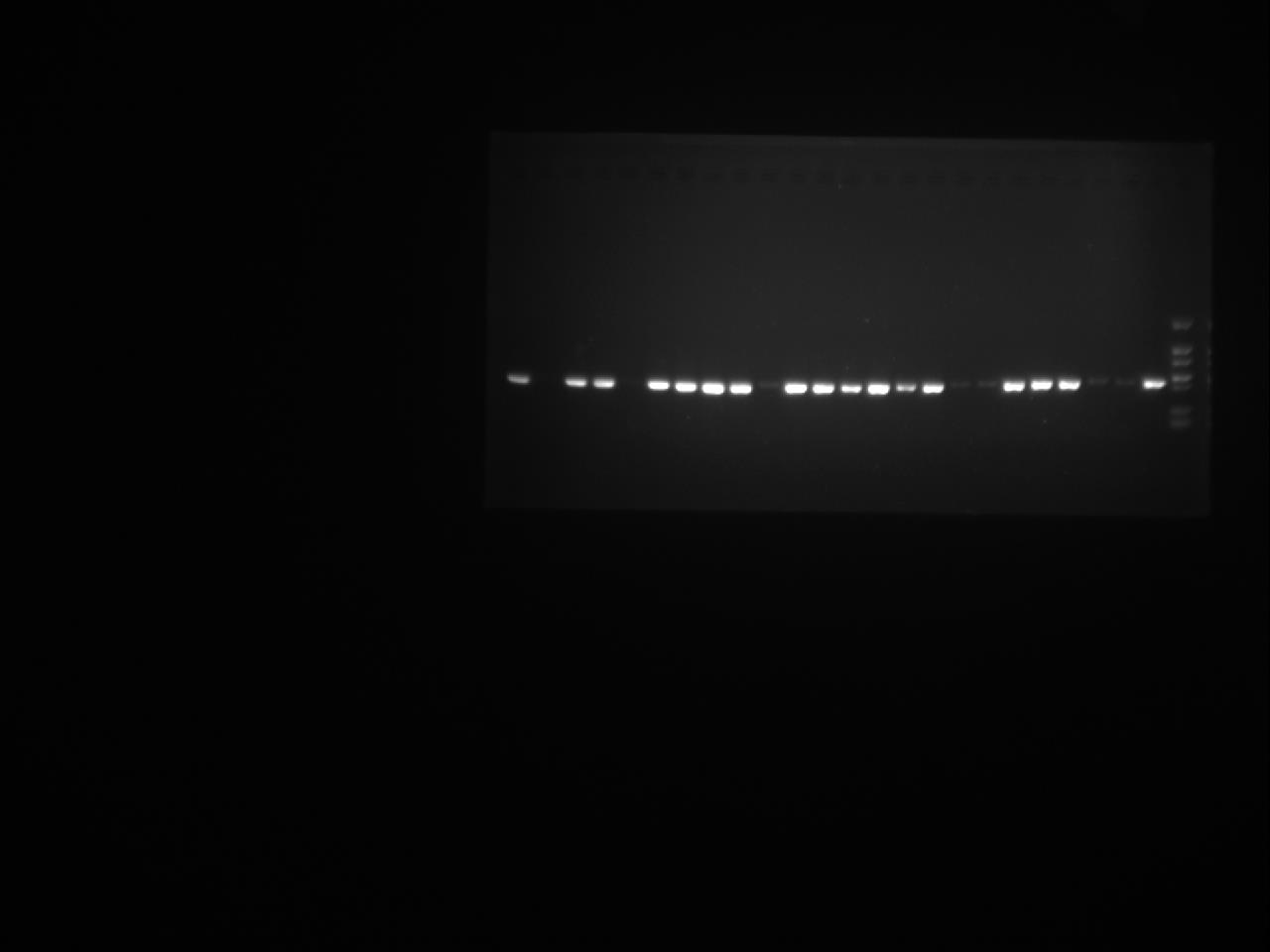

Supplement: Supplemental Information 1 [file peerj-12-17648-s001.zip › original image/Figure 3.Identification of MAT genotypes of single-conidium isolated from F1 strains by PCR amplification/jyt-1/jyt-1(b).jpg]

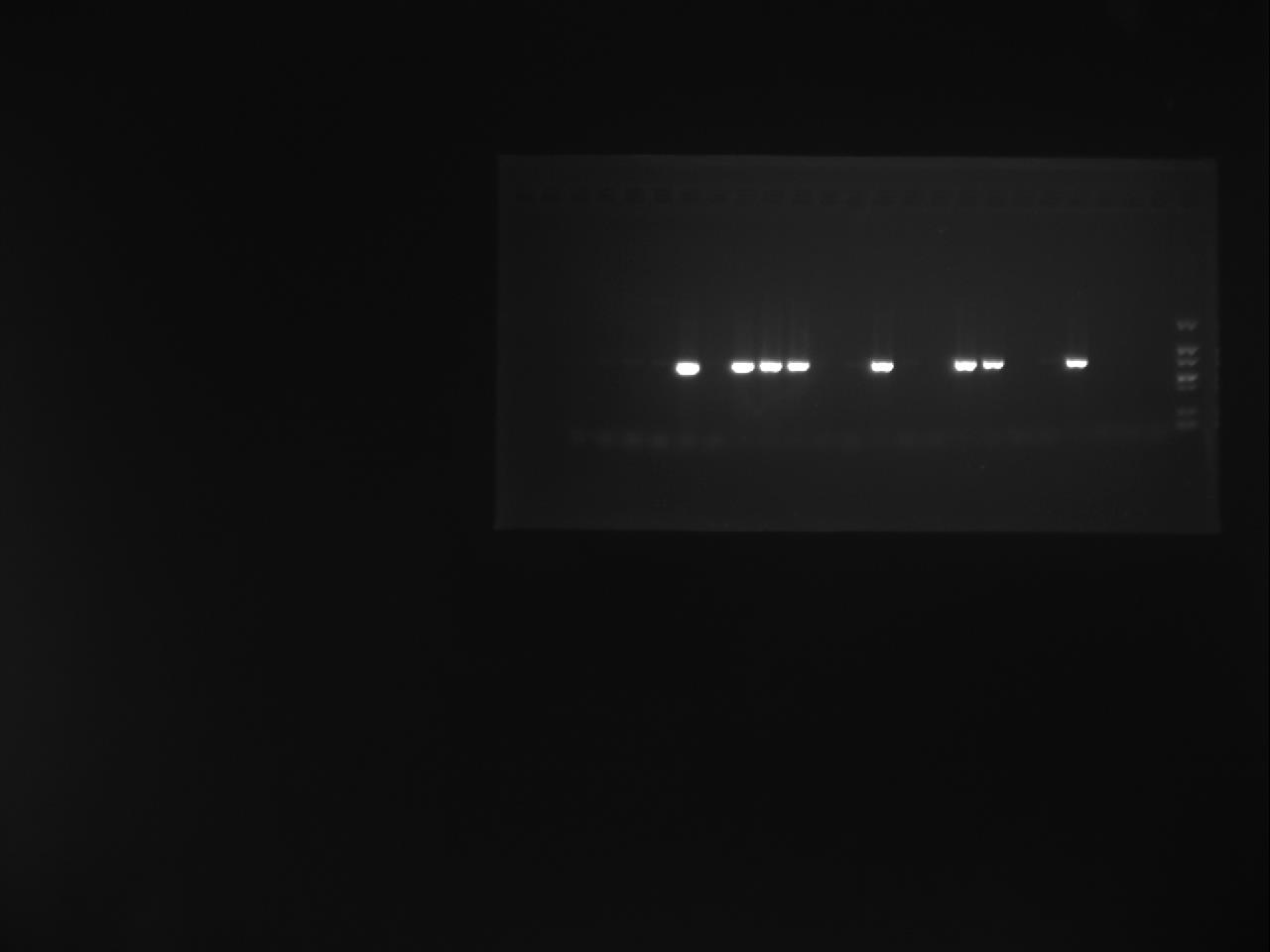

Supplement: Supplemental Information 1 [file peerj-12-17648-s001.zip › original image/Figure 3.Identification of MAT genotypes of single-conidium isolated from F1 strains by PCR amplification/jyt-1/jyt-1(c).jpg]

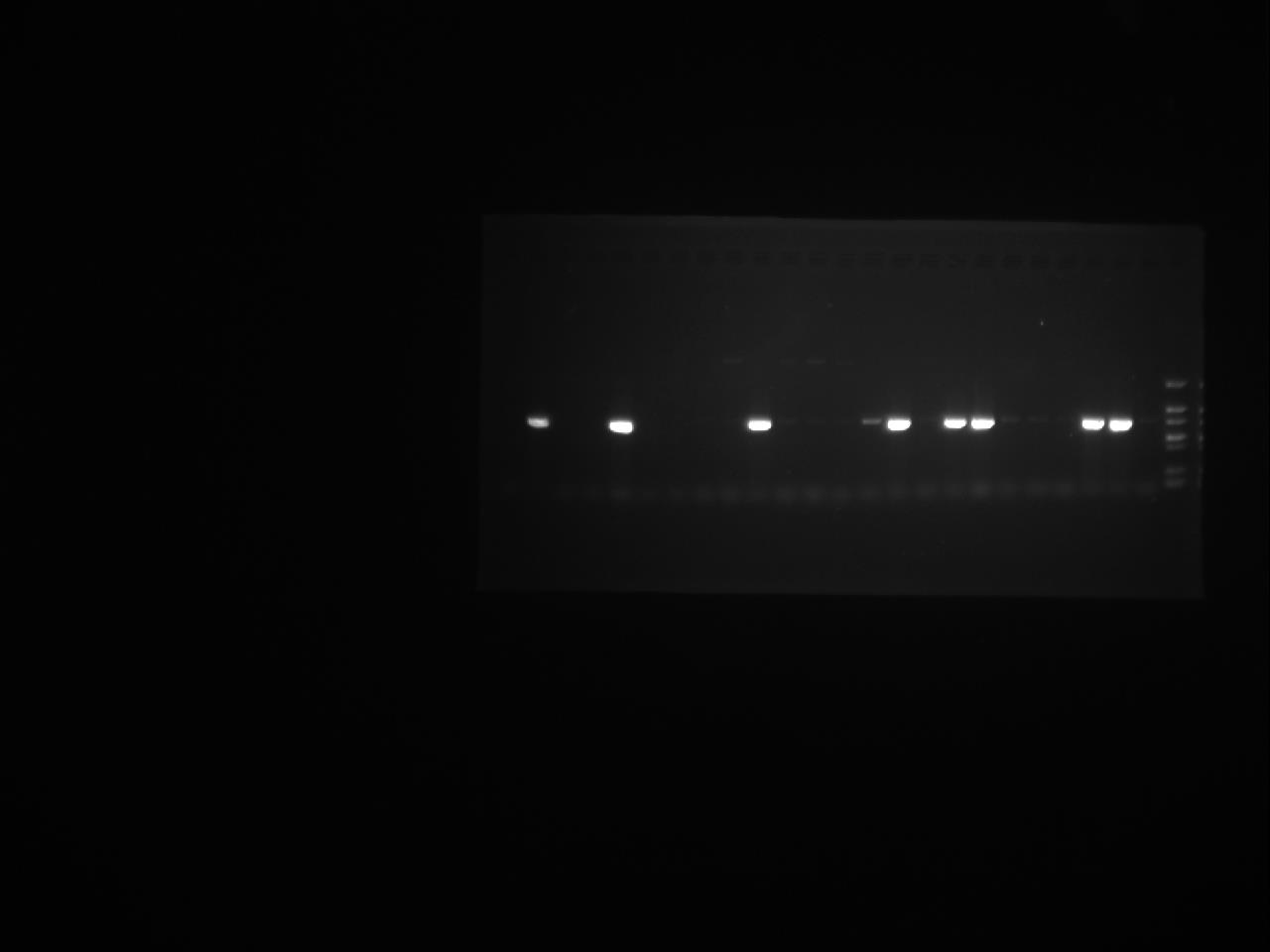

Supplement: Supplemental Information 1 [file peerj-12-17648-s001.zip › original image/Figure 3.Identification of MAT genotypes of single-conidium isolated from F1 strains by PCR amplification/jyt-1/jyt-1(d).jpg]

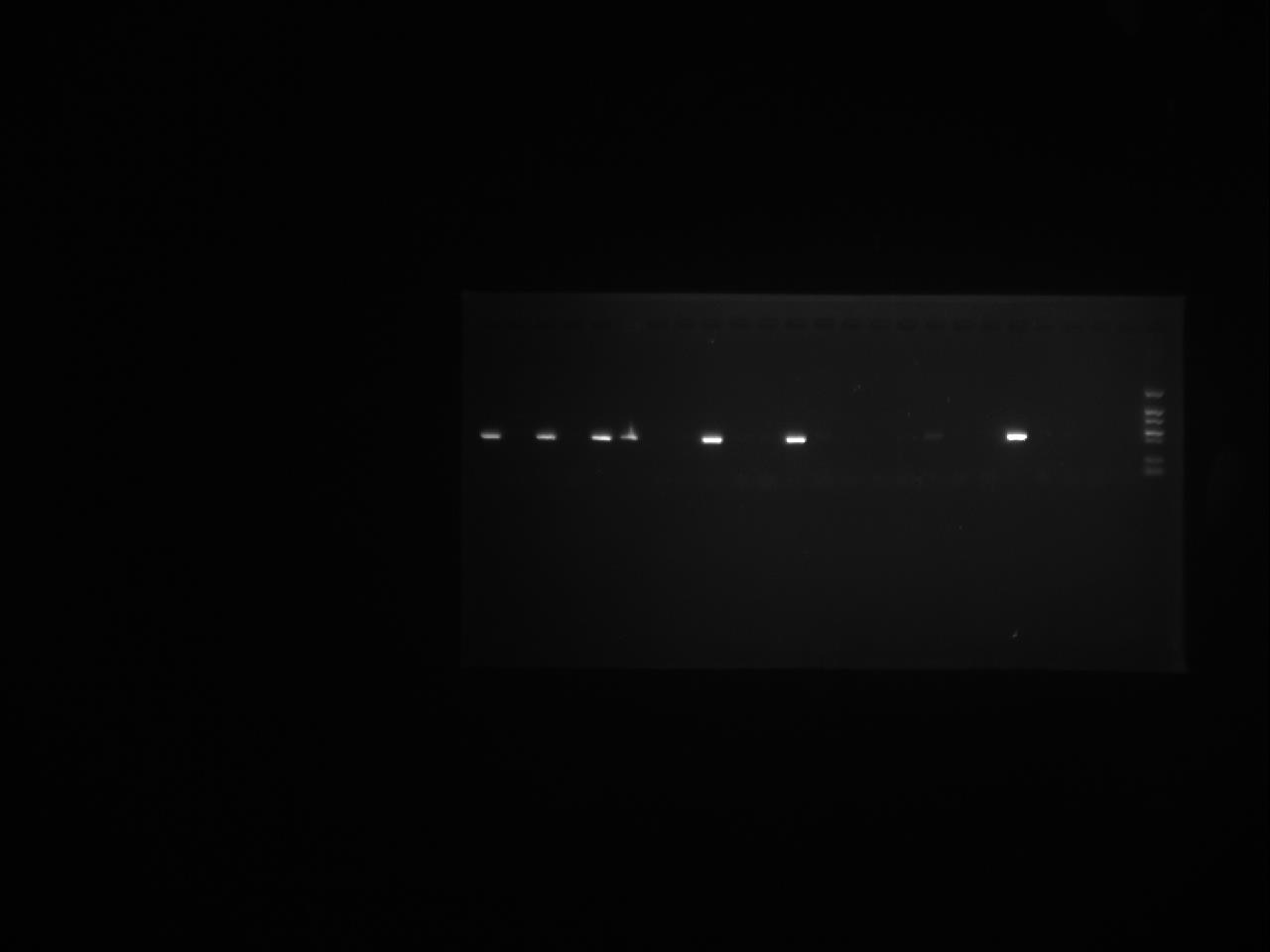

Supplement: Supplemental Information 1 [file peerj-12-17648-s001.zip › original image/Figure 3.Identification of MAT genotypes of single-conidium isolated from F1 strains by PCR amplification/ms-5/ms-5(a).jpg]

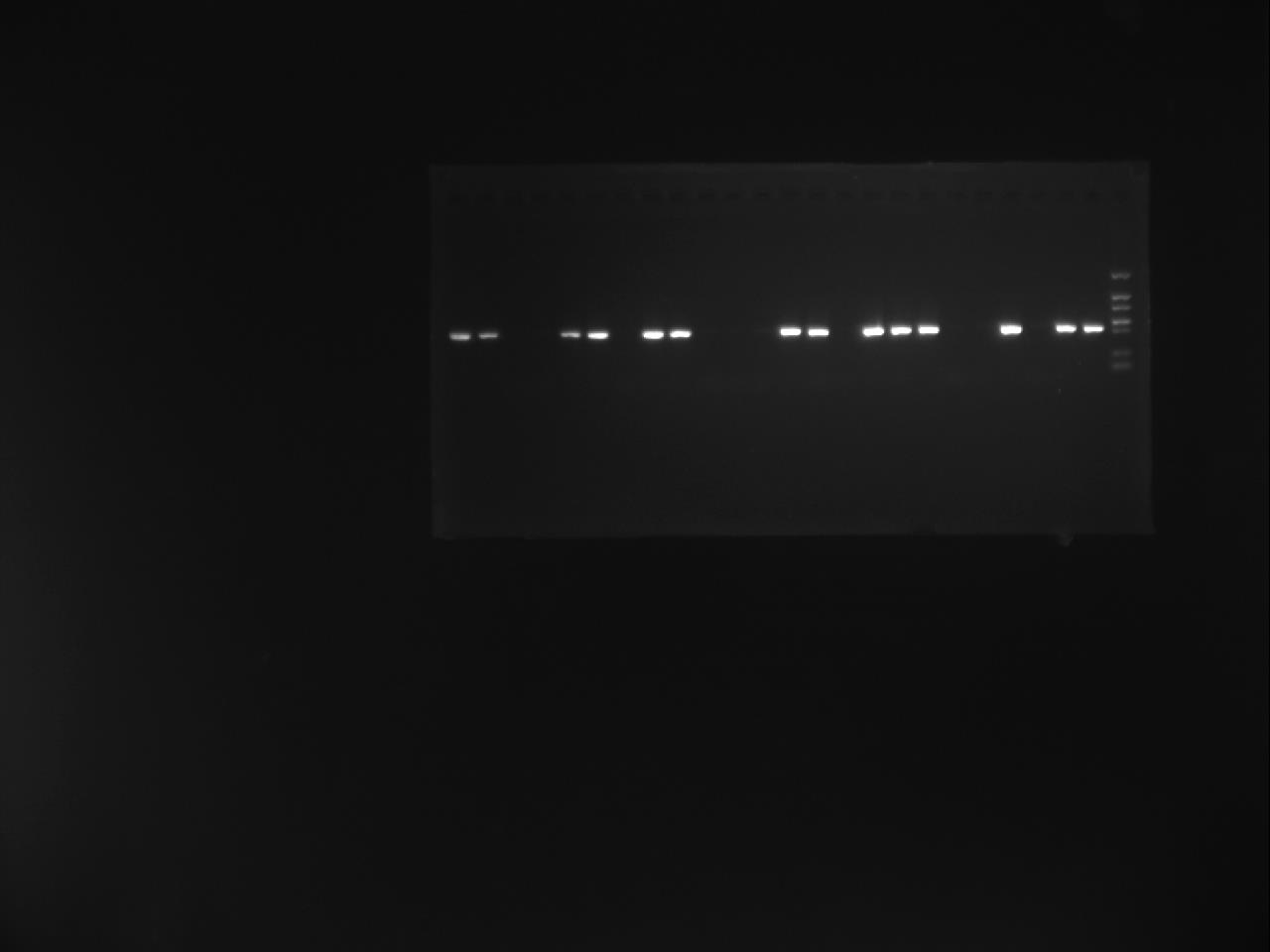

Supplement: Supplemental Information 1 [file peerj-12-17648-s001.zip › original image/Figure 3.Identification of MAT genotypes of single-conidium isolated from F1 strains by PCR amplification/ms-5/ms-5(b).jpg]

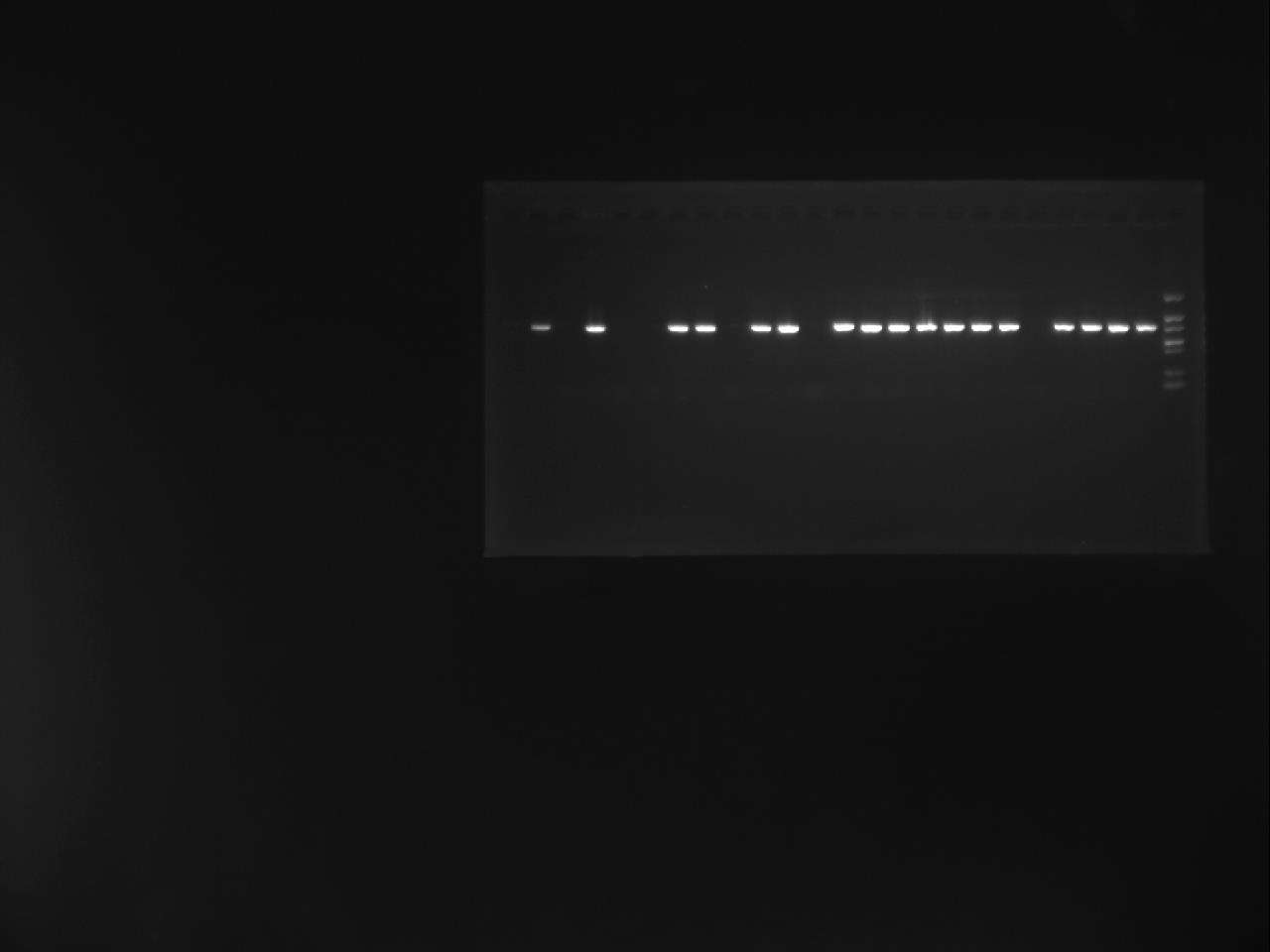

Supplement: Supplemental Information 1 [file peerj-12-17648-s001.zip › original image/Figure 3.Identification of MAT genotypes of single-conidium isolated from F1 strains by PCR amplification/ms-5/ms-5(c).jpg]

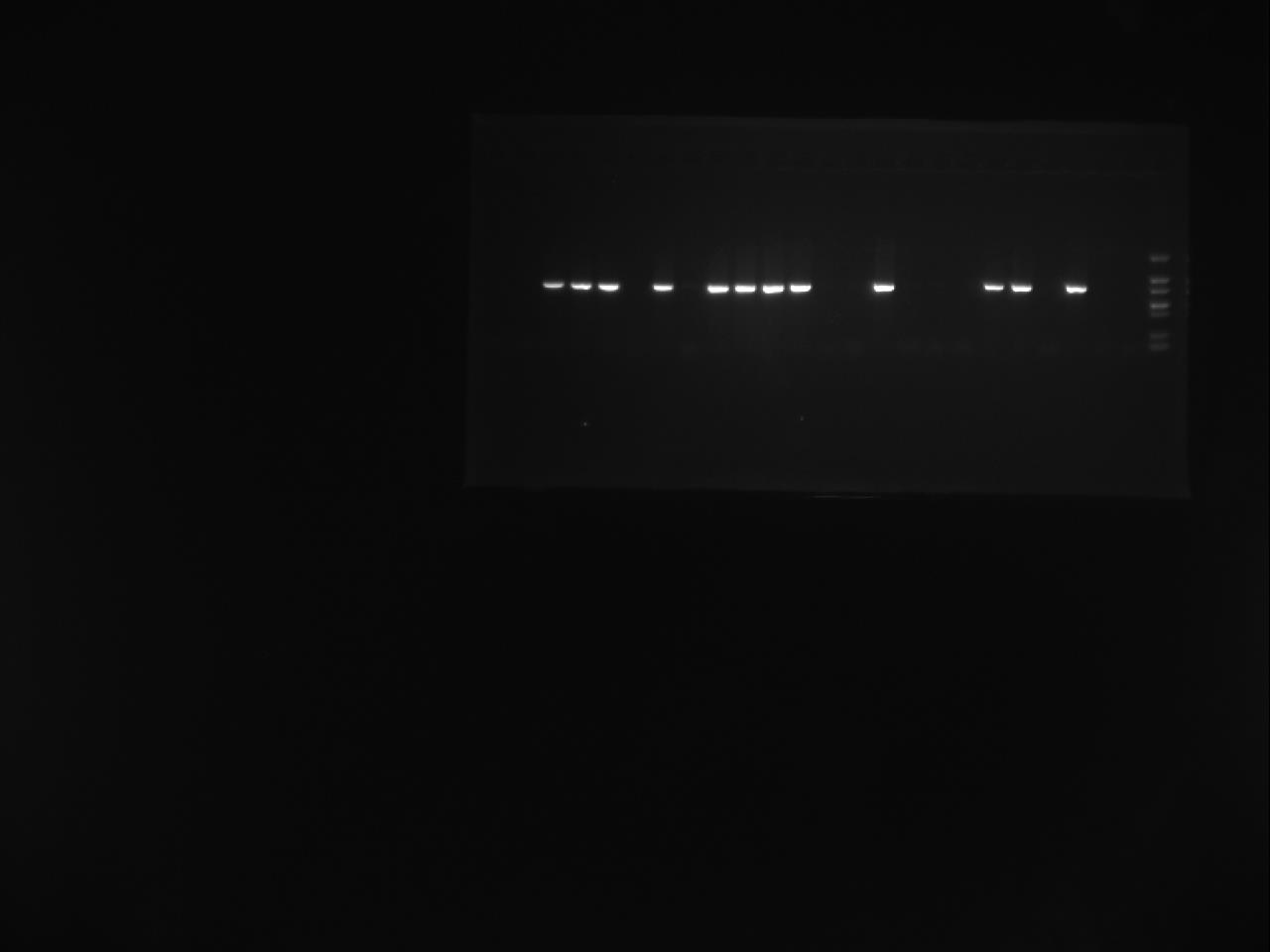

Supplement: Supplemental Information 1 [file peerj-12-17648-s001.zip › original image/Figure 3.Identification of MAT genotypes of single-conidium isolated from F1 strains by PCR amplification/ms-5/ms-5(d).jpg]

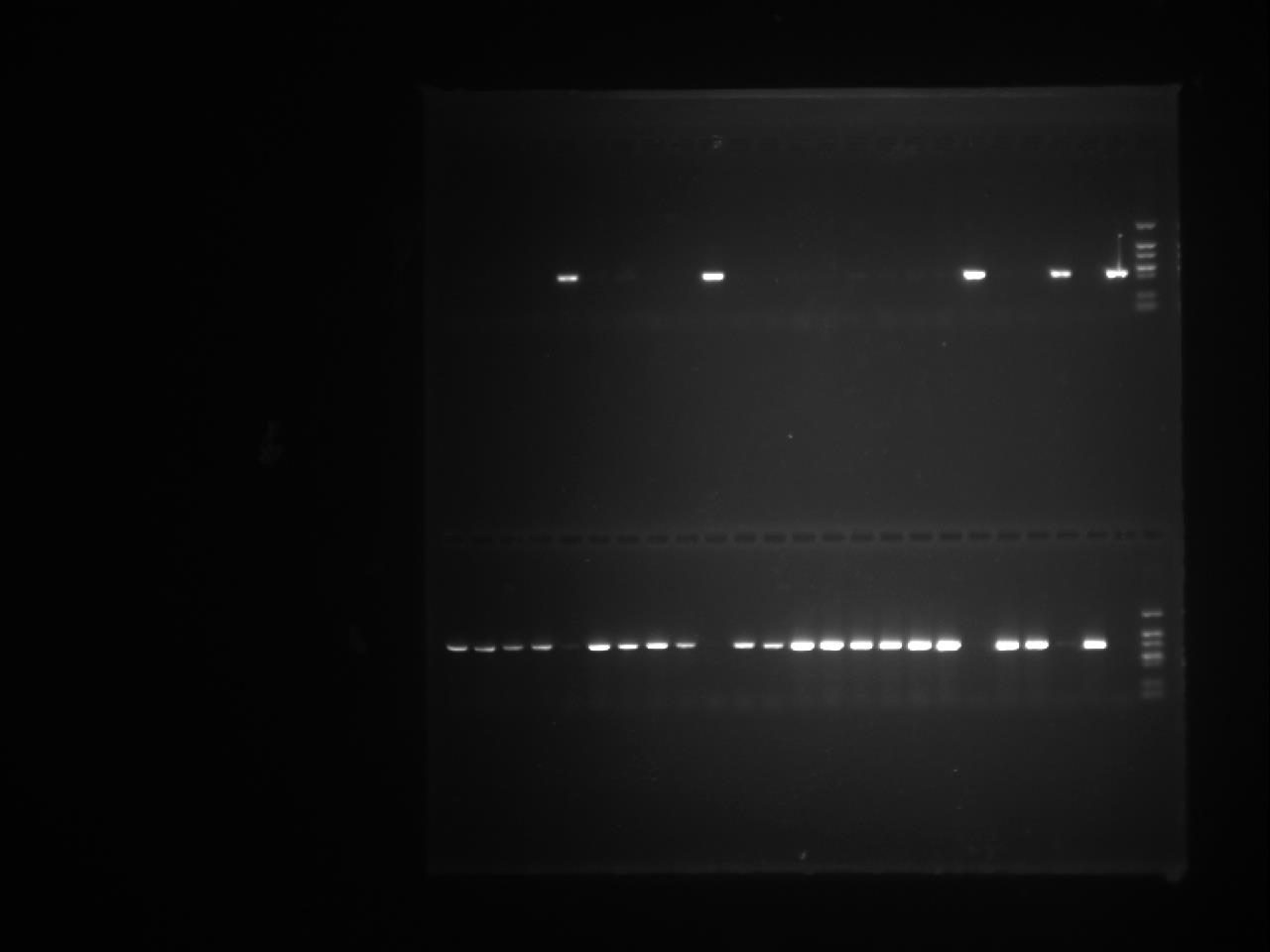

Supplement: Supplemental Information 1 [file peerj-12-17648-s001.zip › original image/Figure 3.Identification of MAT genotypes of single-conidium isolated from F1 strains by PCR amplification/xf-1/xf-1(a,c).jpg]

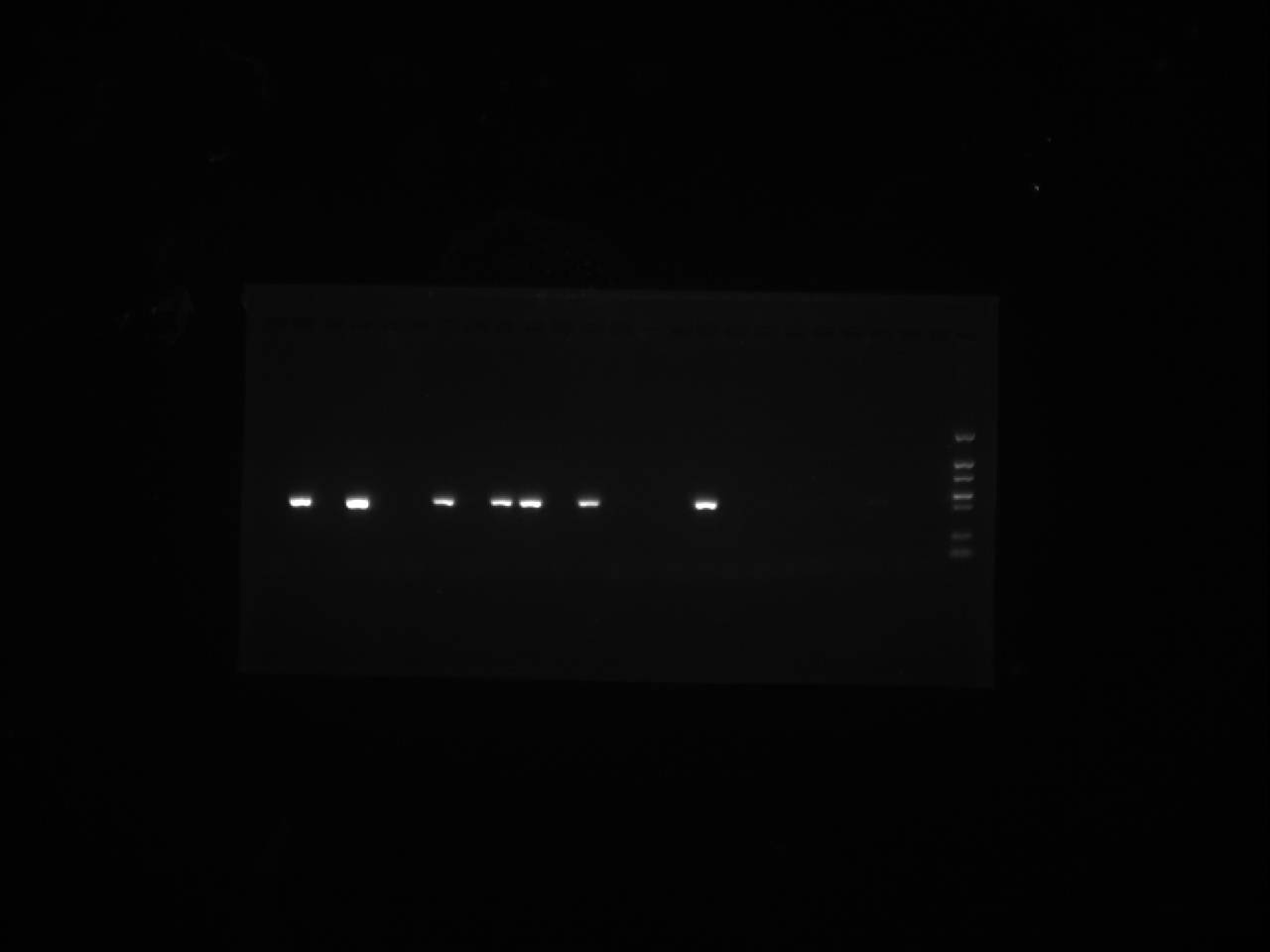

Supplement: Supplemental Information 1 [file peerj-12-17648-s001.zip › original image/Figure 3.Identification of MAT genotypes of single-conidium isolated from F1 strains by PCR amplification/xf-1/xf-1(b).jpg]

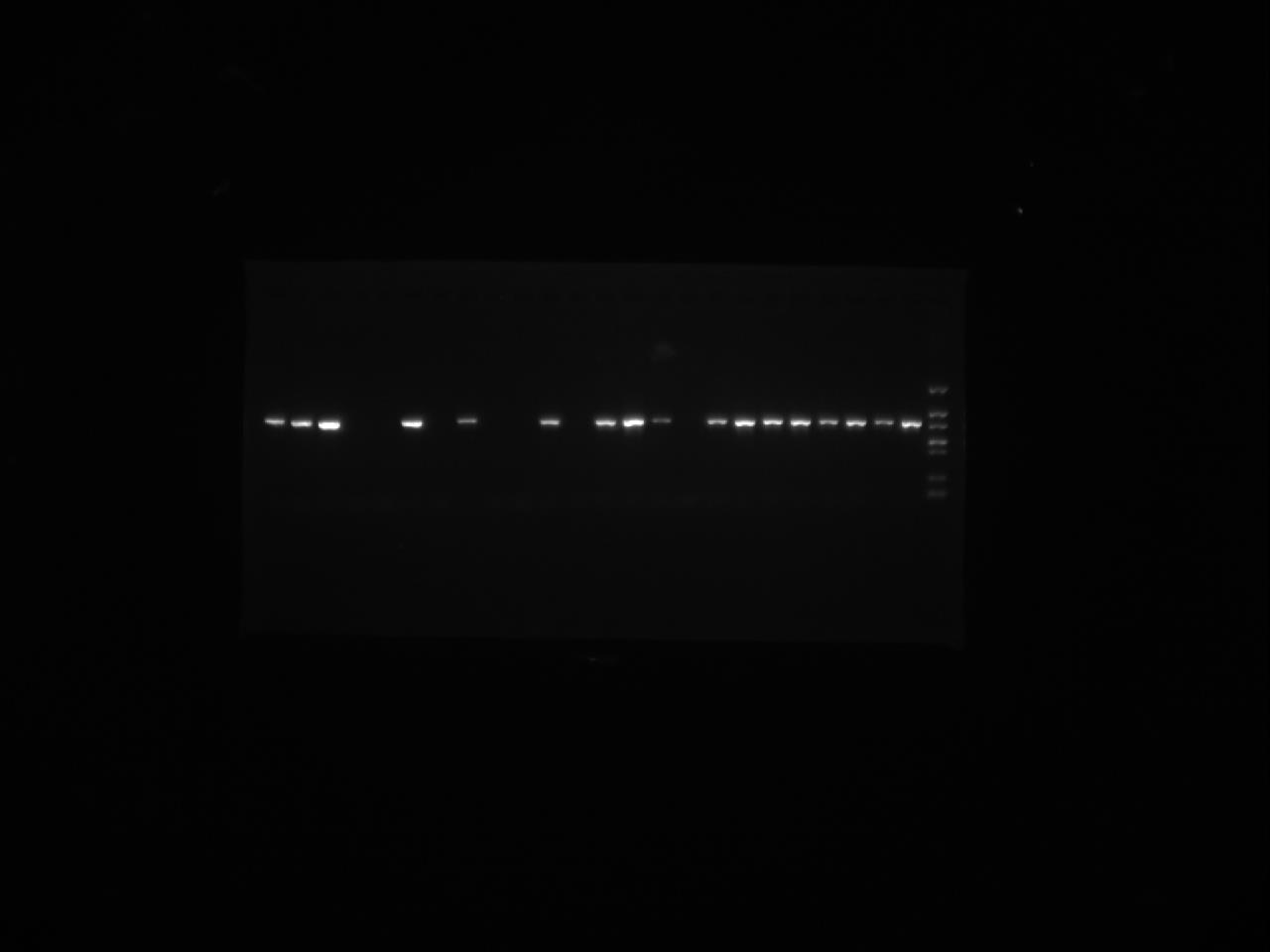

Supplement: Supplemental Information 1 [file peerj-12-17648-s001.zip › original image/Figure 3.Identification of MAT genotypes of single-conidium isolated from F1 strains by PCR amplification/xf-1/xf-1(d).jpg]

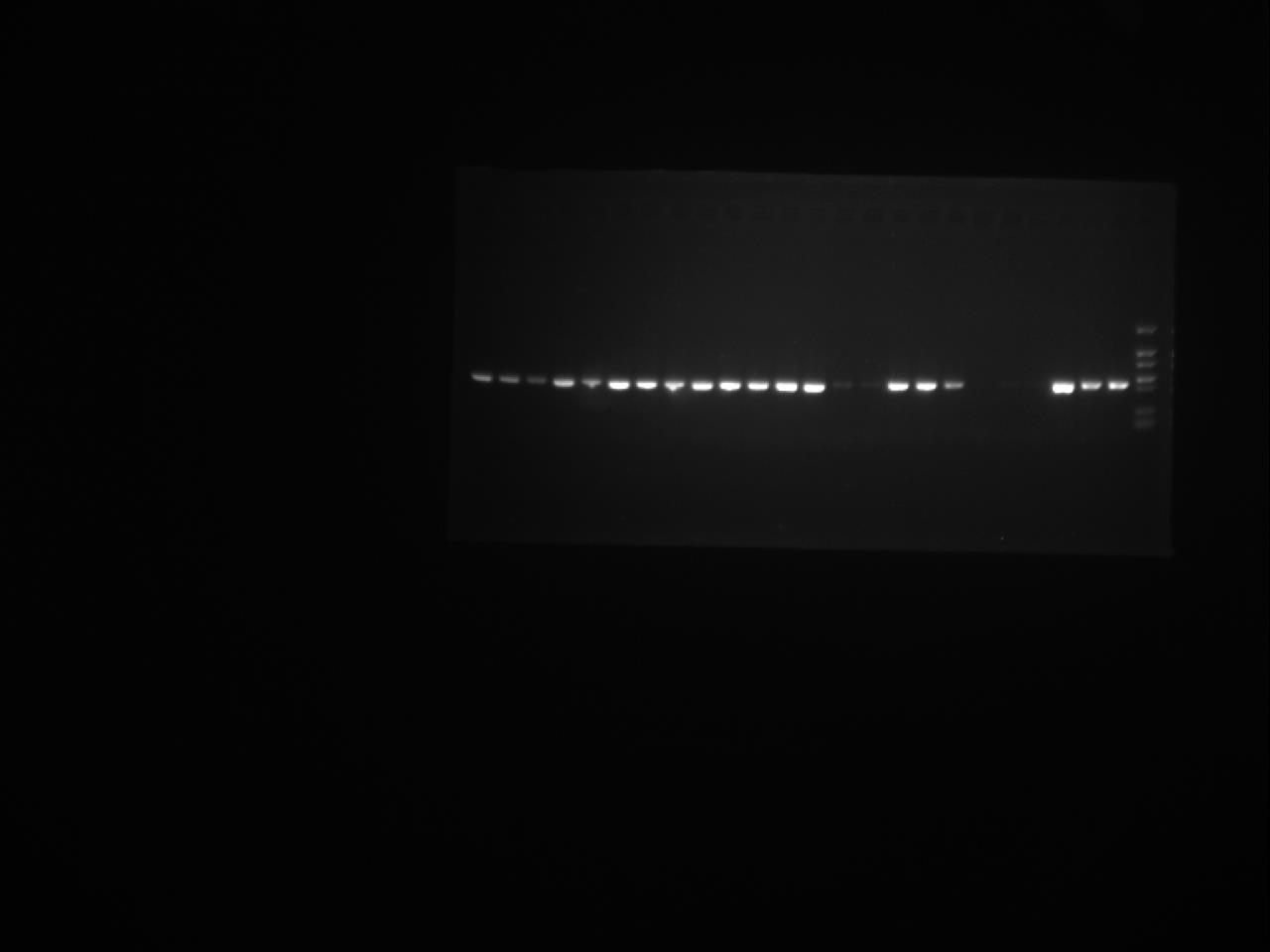

Supplement: Supplemental Information 1 [file peerj-12-17648-s001.zip › original image/Figure 3.Identification of MAT genotypes of single-conidium isolated from F1 strains by PCR amplification/xm-1/xm-1(a).jpg]

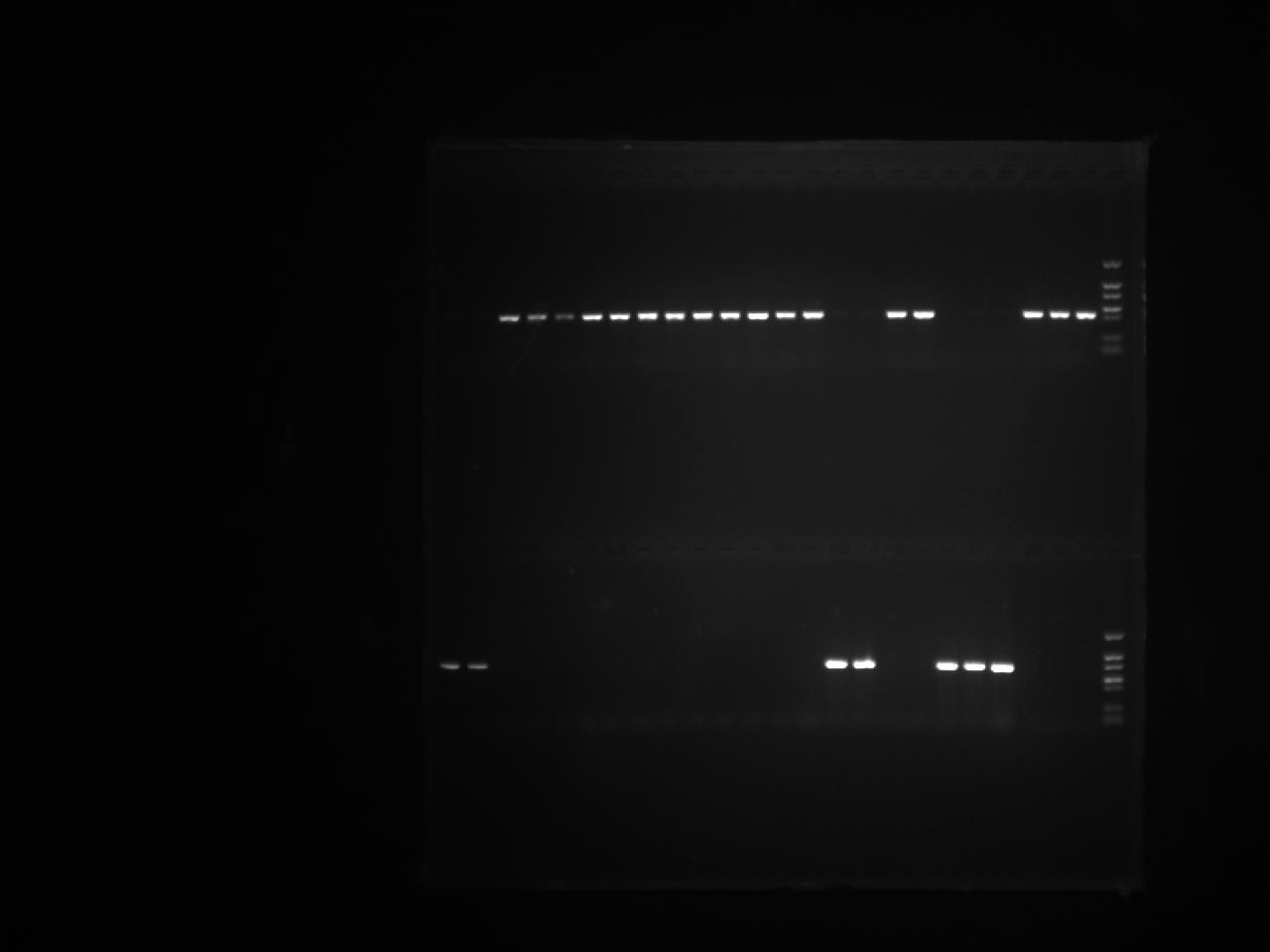

Supplement: Supplemental Information 1 [file peerj-12-17648-s001.zip › original image/Figure 3.Identification of MAT genotypes of single-conidium isolated from F1 strains by PCR amplification/xm-1/xm-1(b,d).jpg]

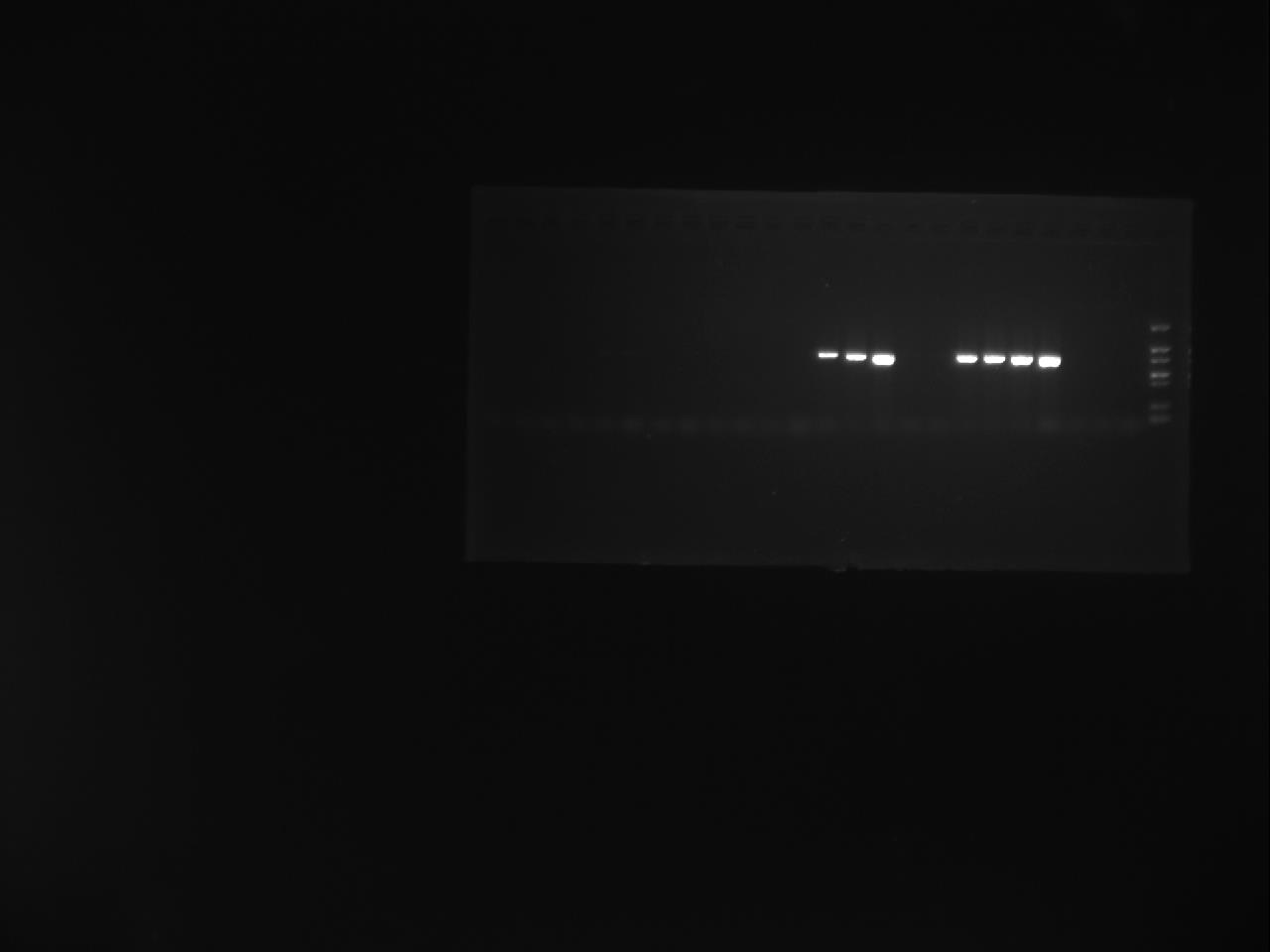

Supplement: Supplemental Information 1 [file peerj-12-17648-s001.zip › original image/Figure 3.Identification of MAT genotypes of single-conidium isolated from F1 strains by PCR amplification/xm-1/xm-1(c).jpg]

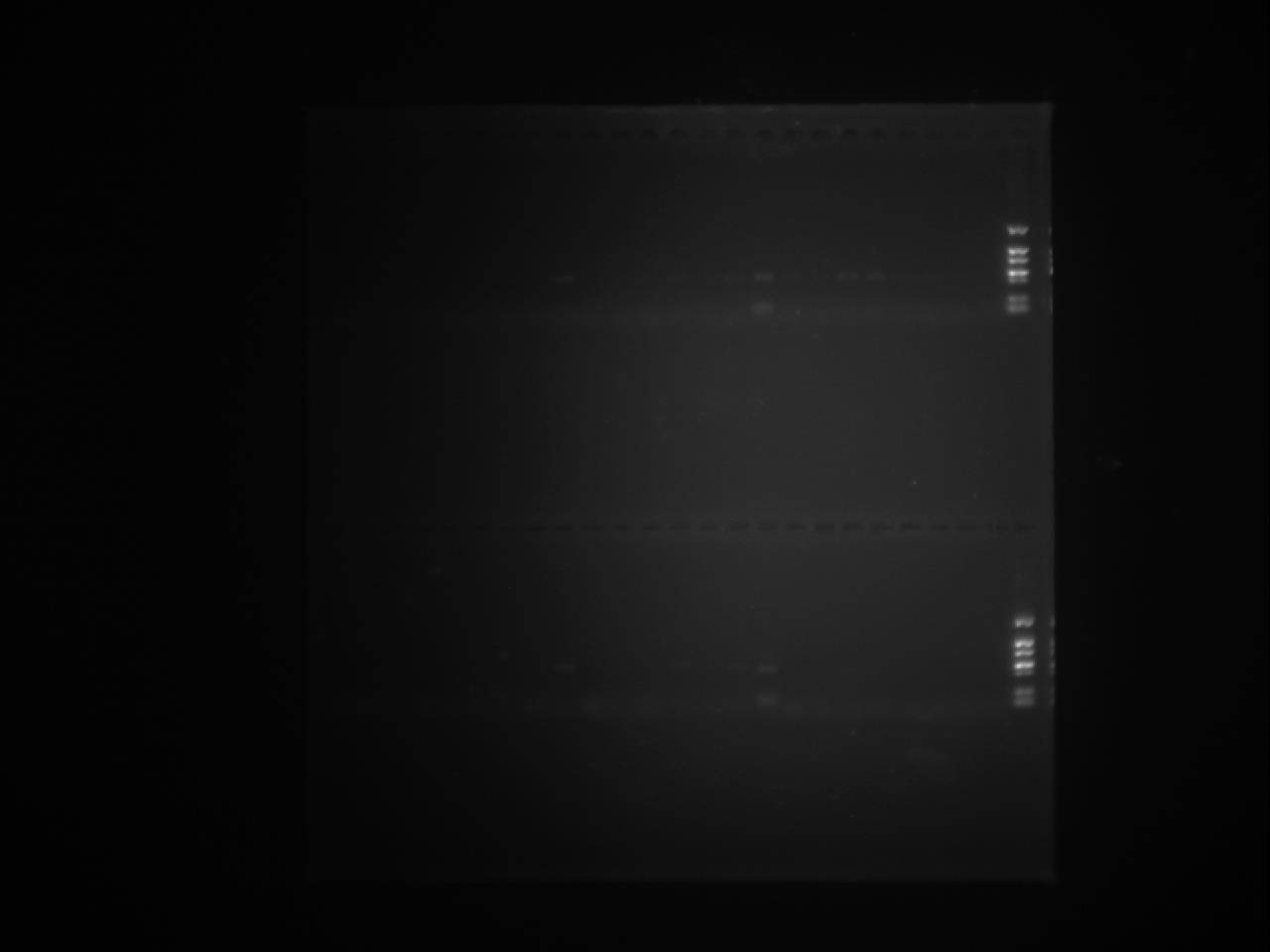

Supplement: Supplemental Information 1 [file peerj-12-17648-s001.zip › original image/Figure 5. Identification of MAT genotypes of single-conidium in degenerate strains by PCR amplification/jb-1/jb-1(a,b).jpg]

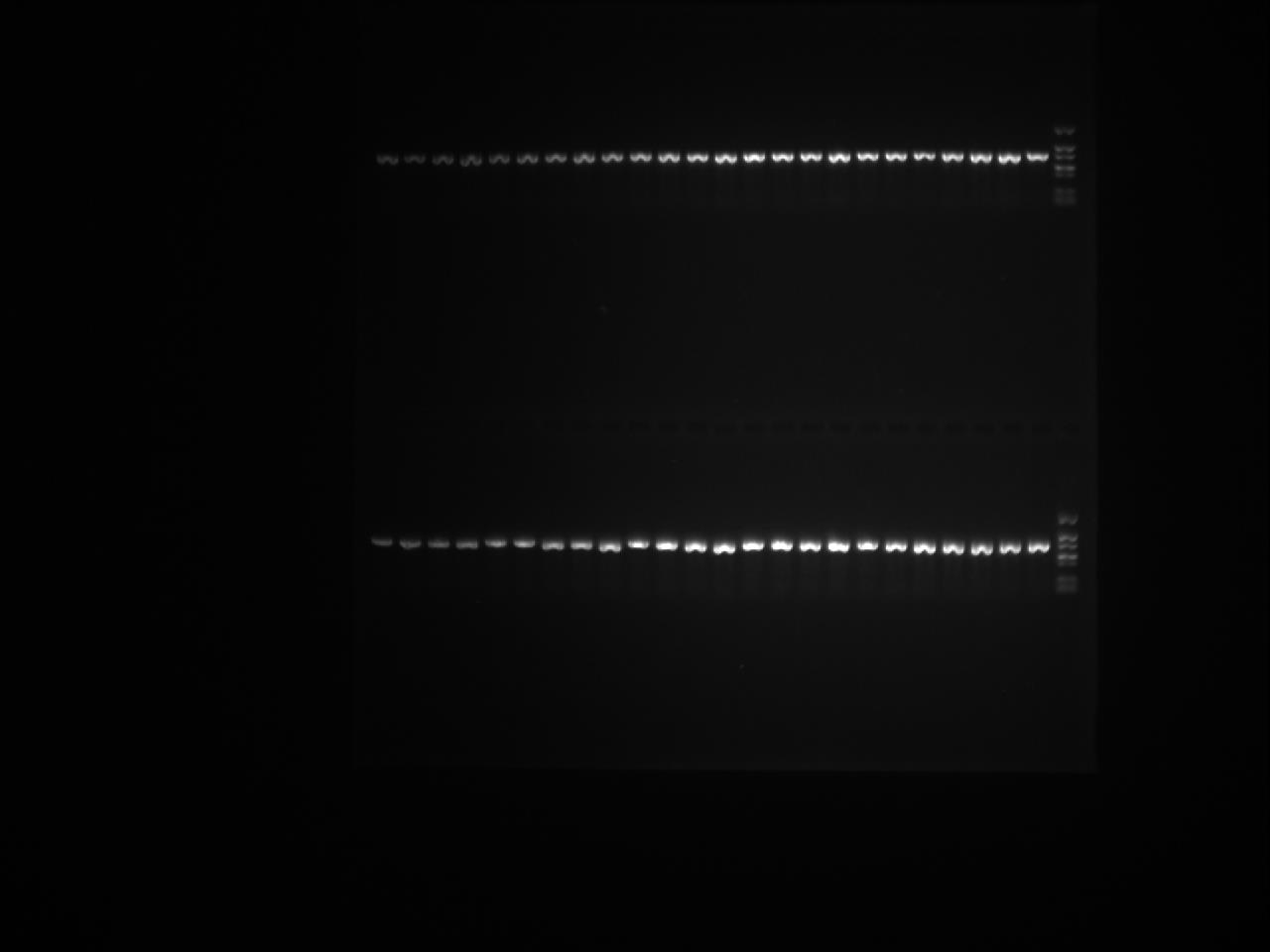

Supplement: Supplemental Information 1 [file peerj-12-17648-s001.zip › original image/Figure 5. Identification of MAT genotypes of single-conidium in degenerate strains by PCR amplification/jb-1/jb-1(c,d).jpg]

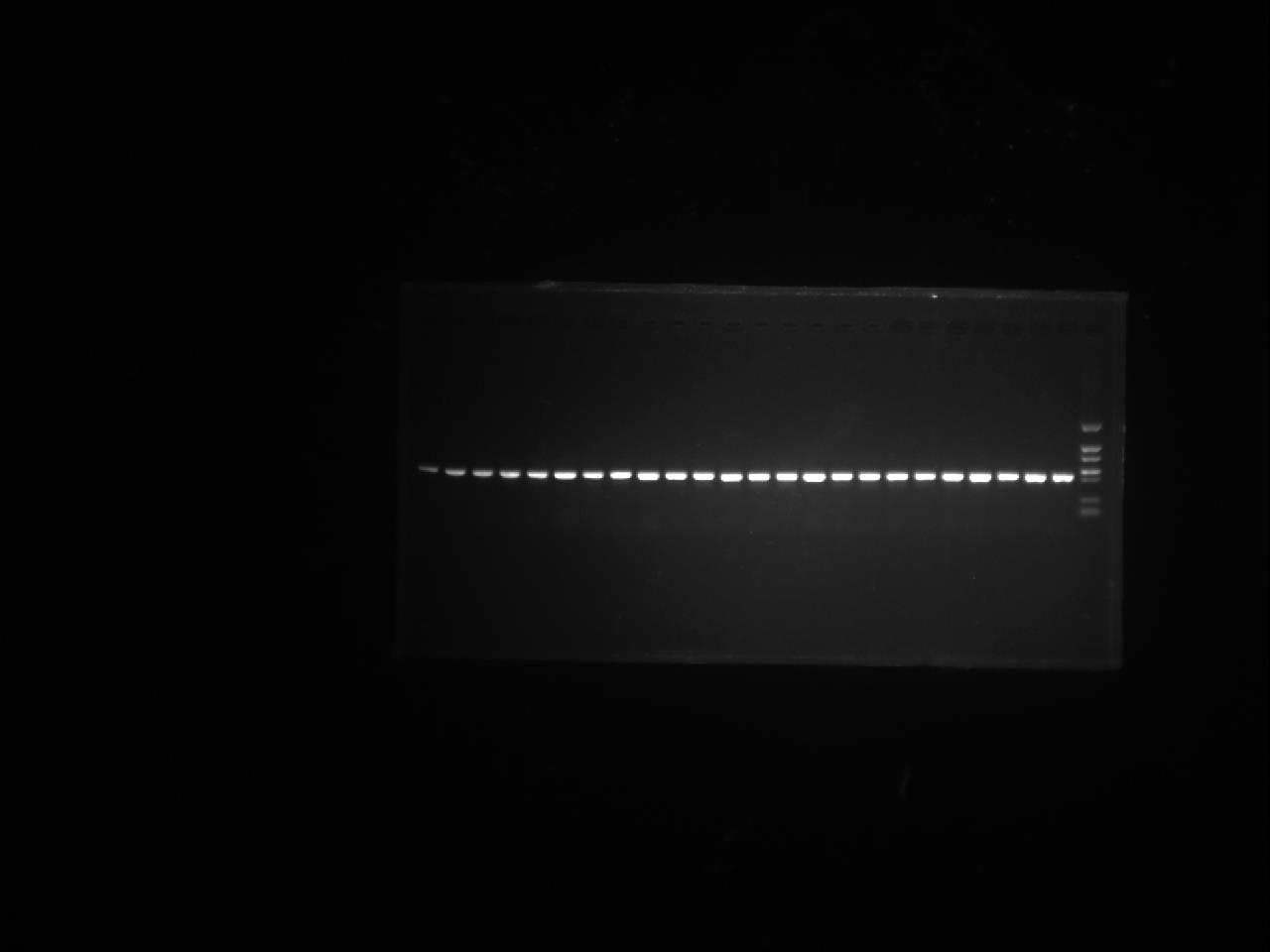

Supplement: Supplemental Information 1 [file peerj-12-17648-s001.zip › original image/Figure 5. Identification of MAT genotypes of single-conidium in degenerate strains by PCR amplification/jyt-1/jyt-1(a).jpg]

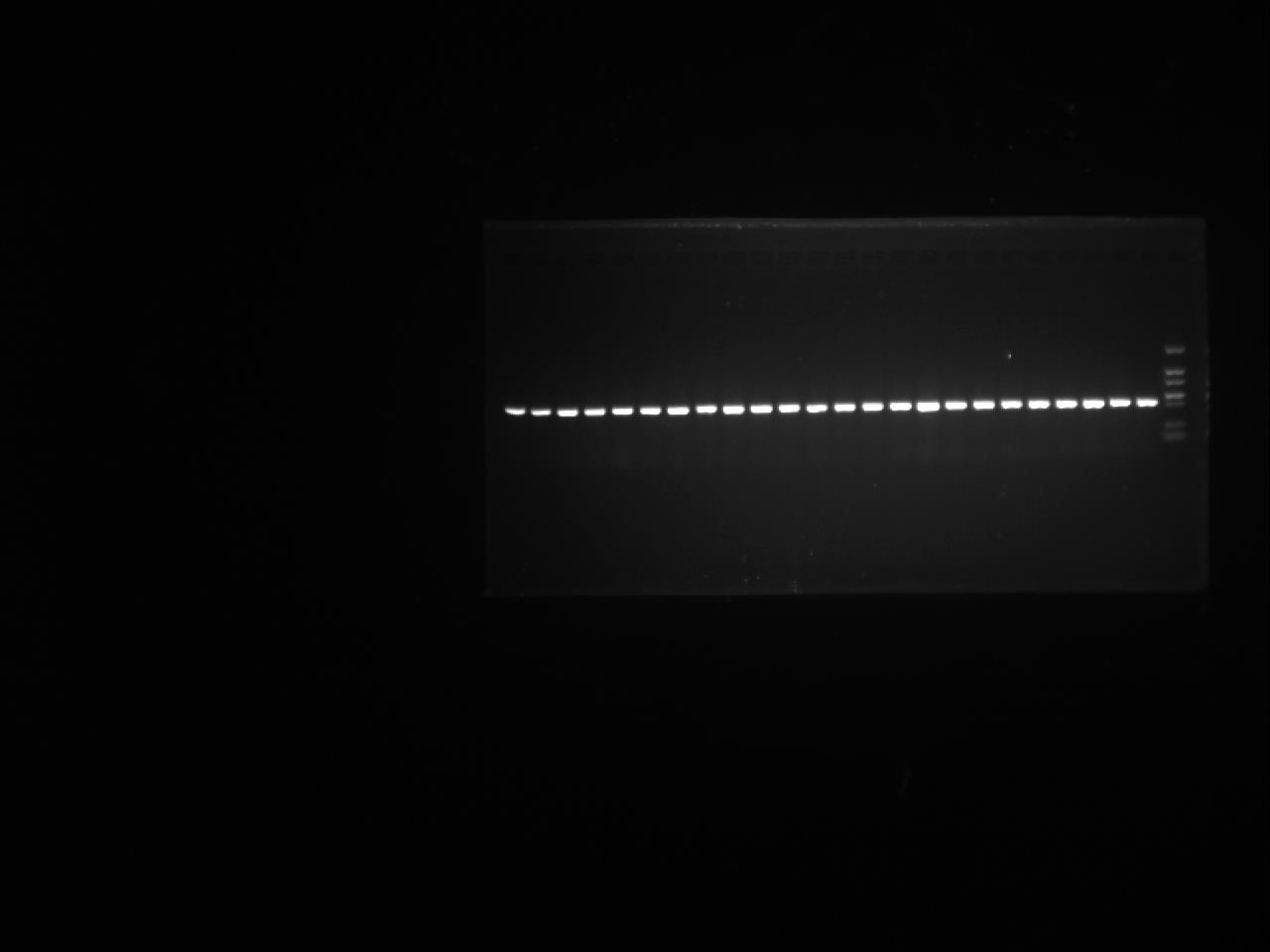

Supplement: Supplemental Information 1 [file peerj-12-17648-s001.zip › original image/Figure 5. Identification of MAT genotypes of single-conidium in degenerate strains by PCR amplification/jyt-1/jyt-1(b).jpg]

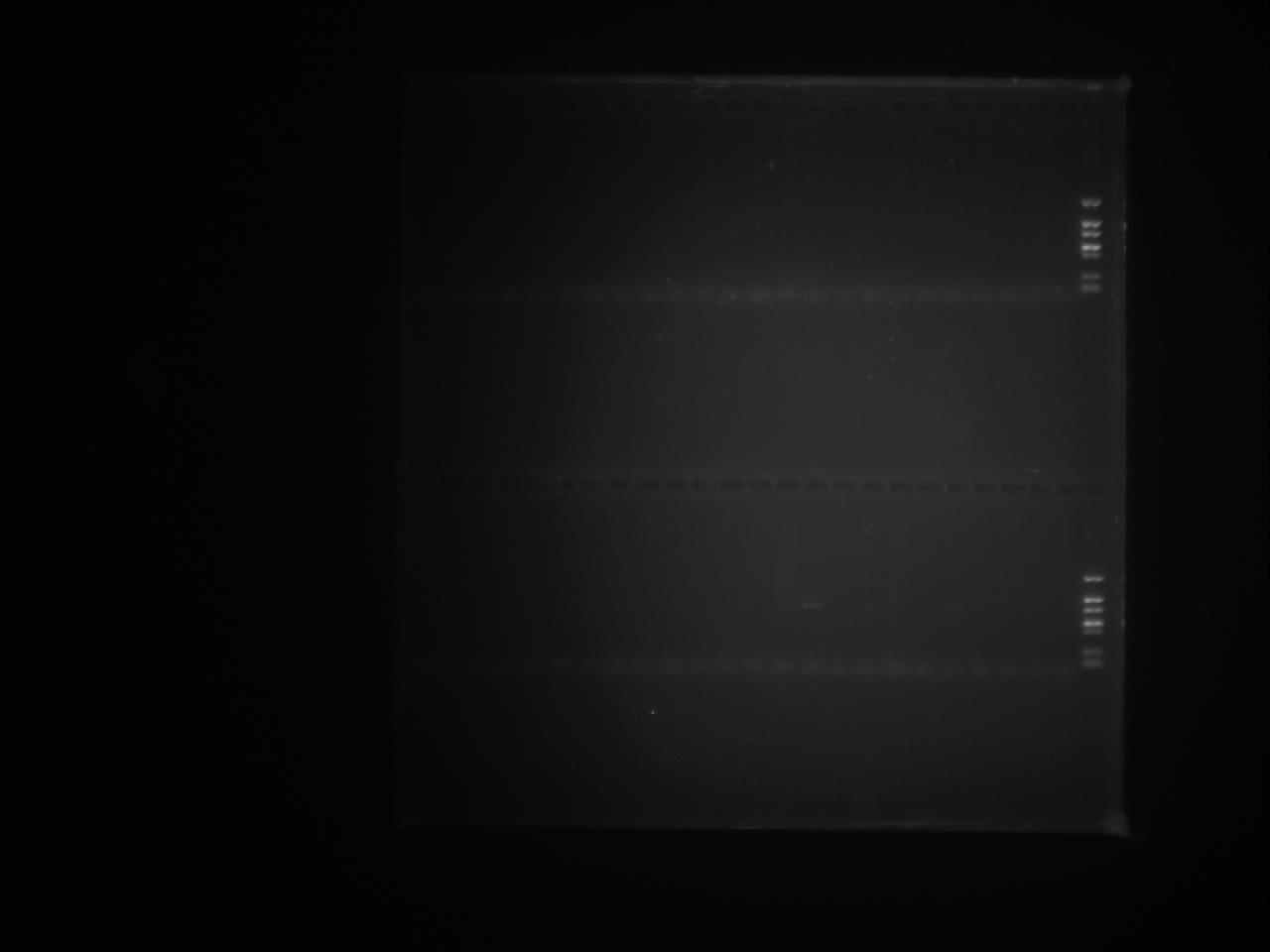

Supplement: Supplemental Information 1 [file peerj-12-17648-s001.zip › original image/Figure 5. Identification of MAT genotypes of single-conidium in degenerate strains by PCR amplification/jyt-1/jyt-1(c,d).jpg]

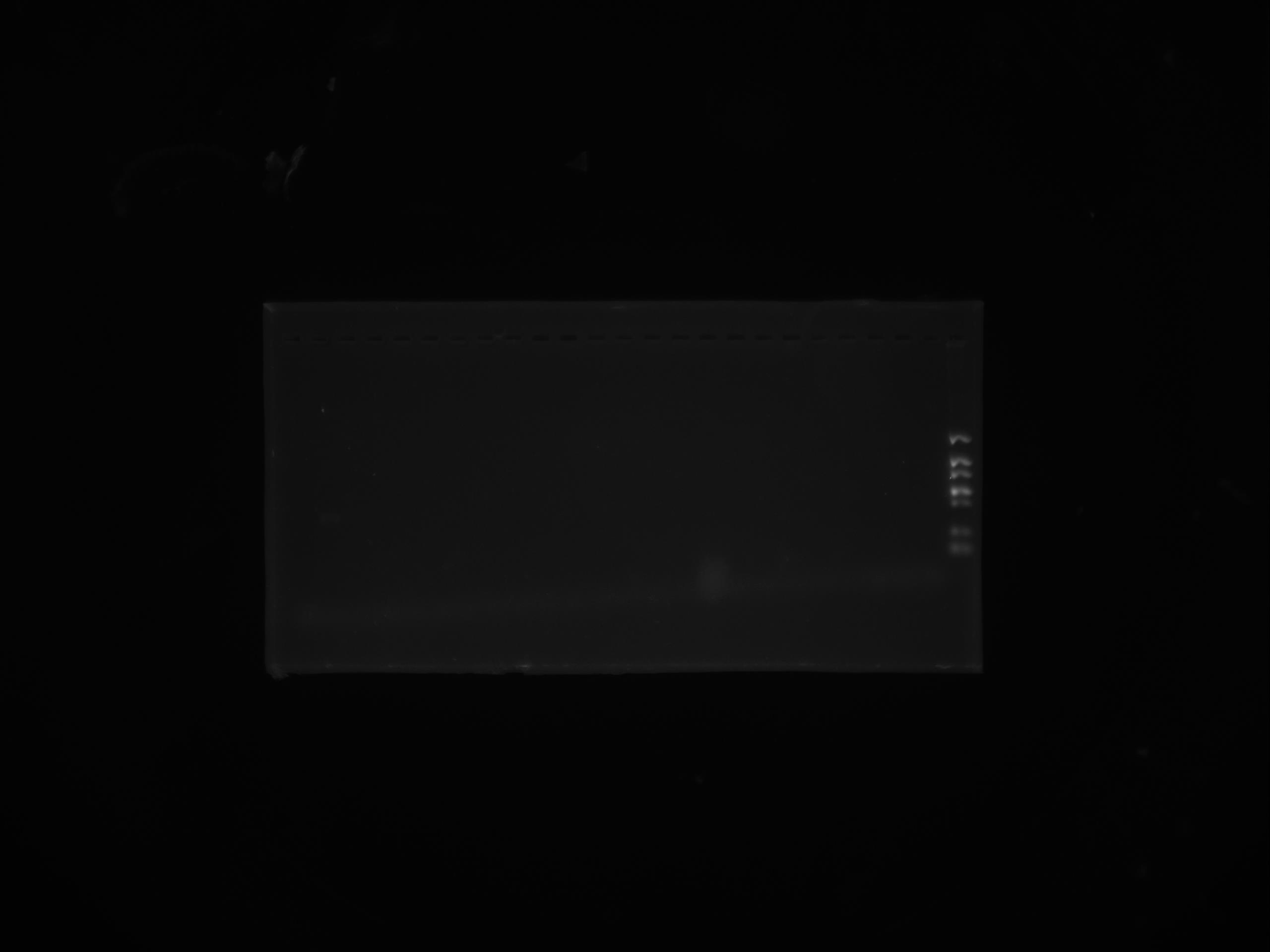

Supplement: Supplemental Information 1 [file peerj-12-17648-s001.zip › original image/Figure 5. Identification of MAT genotypes of single-conidium in degenerate strains by PCR amplification/xf-1/xf-1(a).jpg]

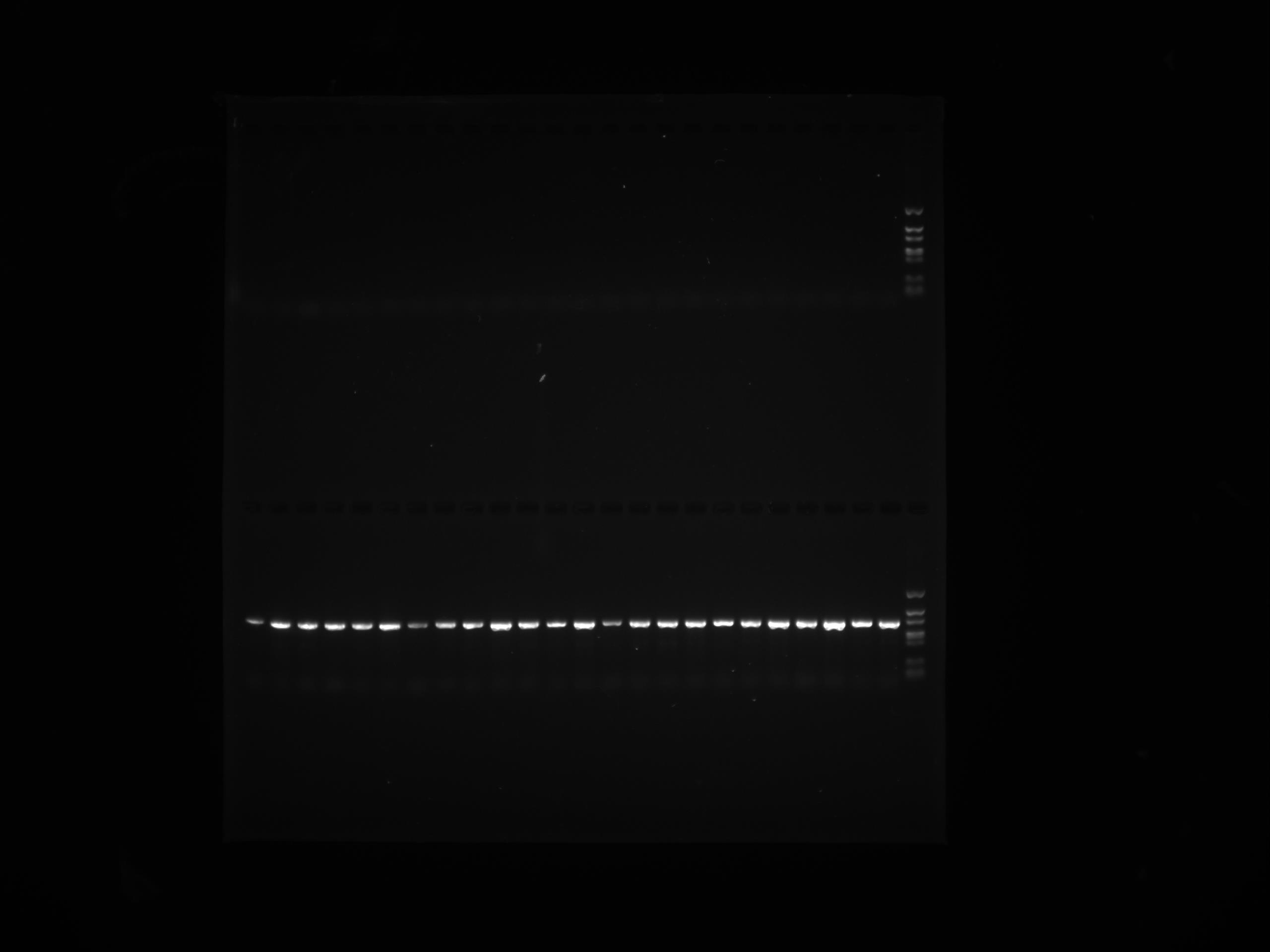

Supplement: Supplemental Information 1 [file peerj-12-17648-s001.zip › original image/Figure 5. Identification of MAT genotypes of single-conidium in degenerate strains by PCR amplification/xf-1/xf-1(b,d).jpg]

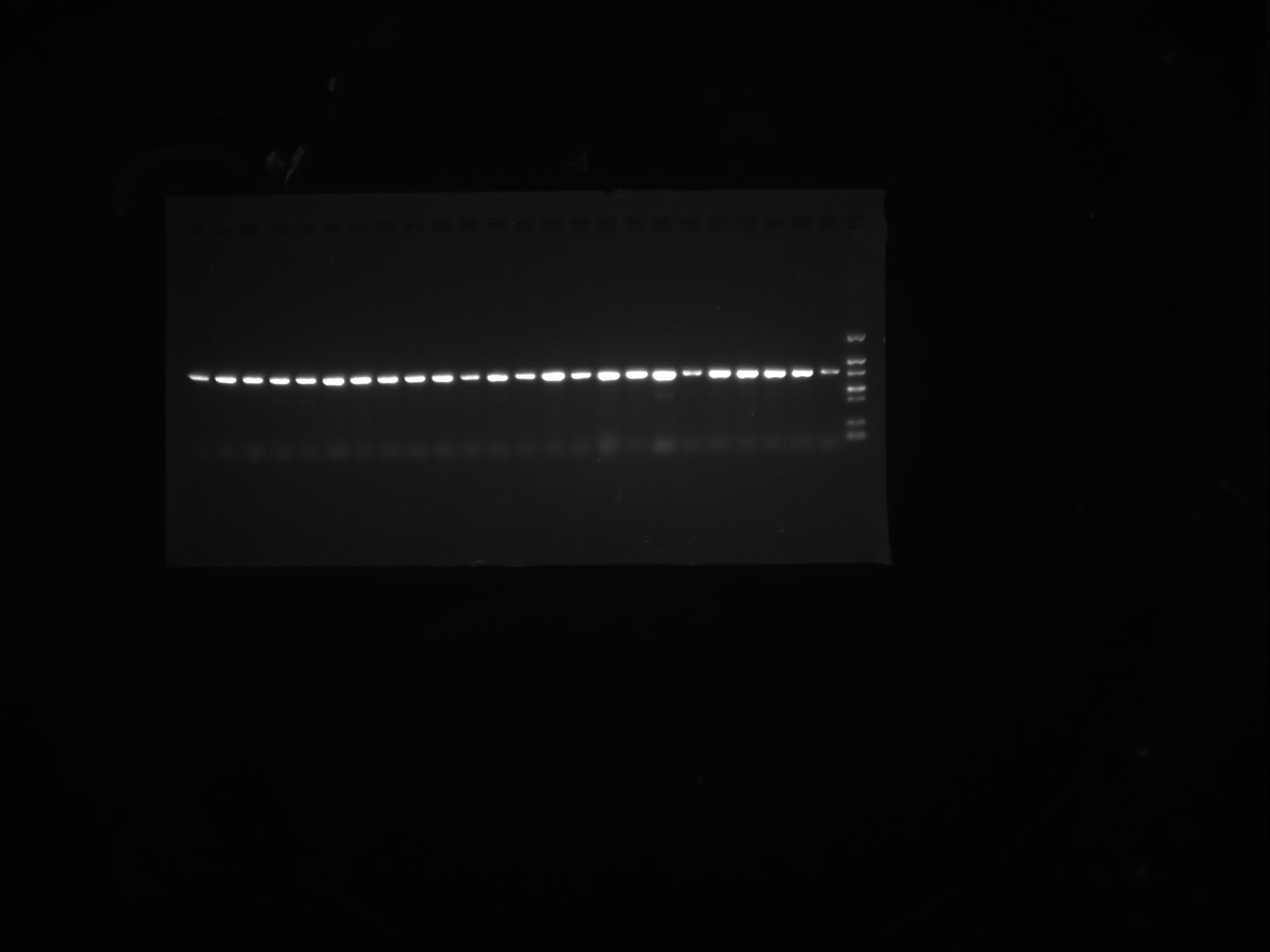

Supplement: Supplemental Information 1 [file peerj-12-17648-s001.zip › original image/Figure 5. Identification of MAT genotypes of single-conidium in degenerate strains by PCR amplification/xf-1/xf-1(c).jpg]

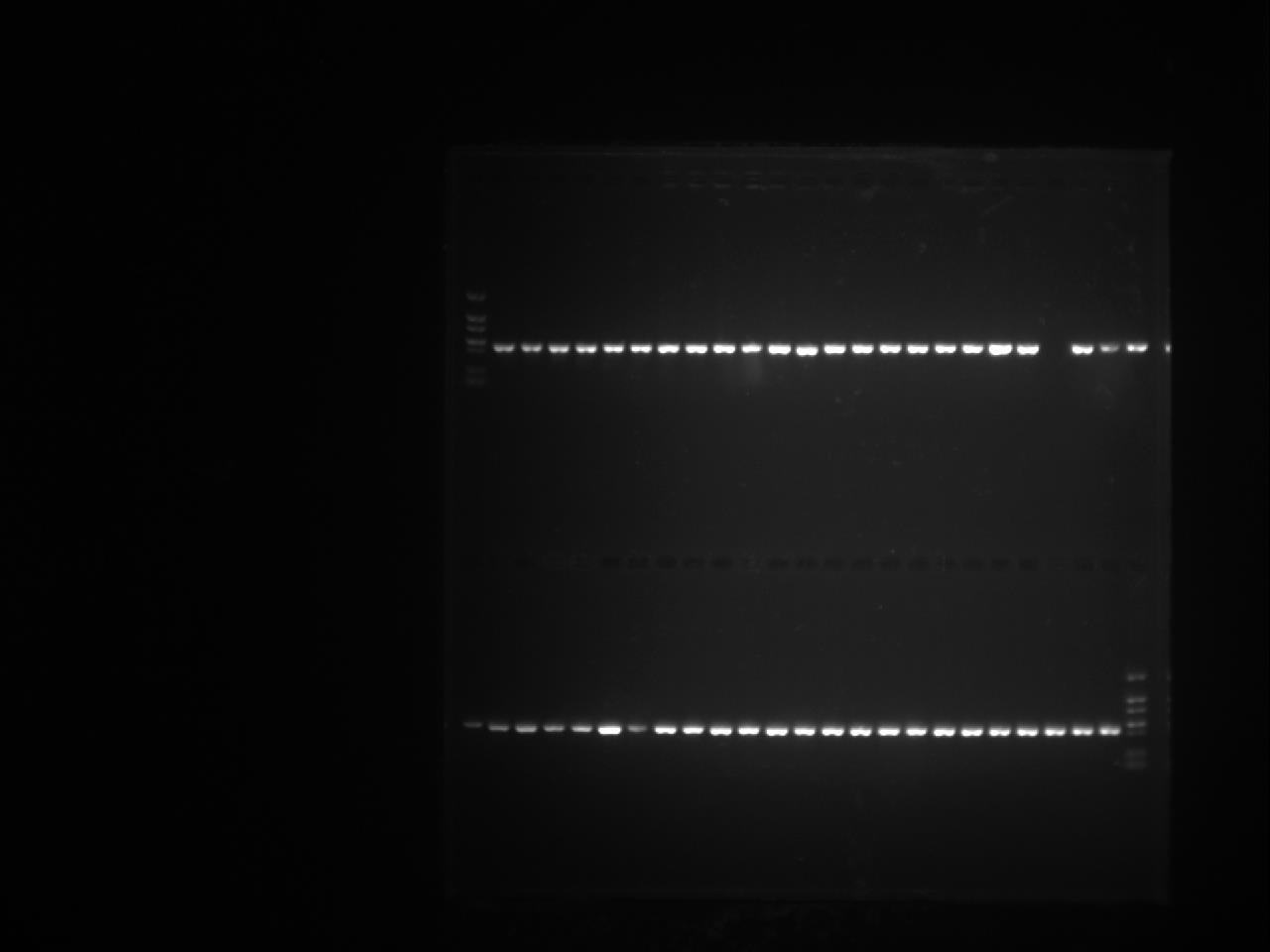

Supplement: Supplemental Information 1 [file peerj-12-17648-s001.zip › original image/Figure 5. Identification of MAT genotypes of single-conidium in degenerate strains by PCR amplification/xm-1/xm-1(b).jpg]

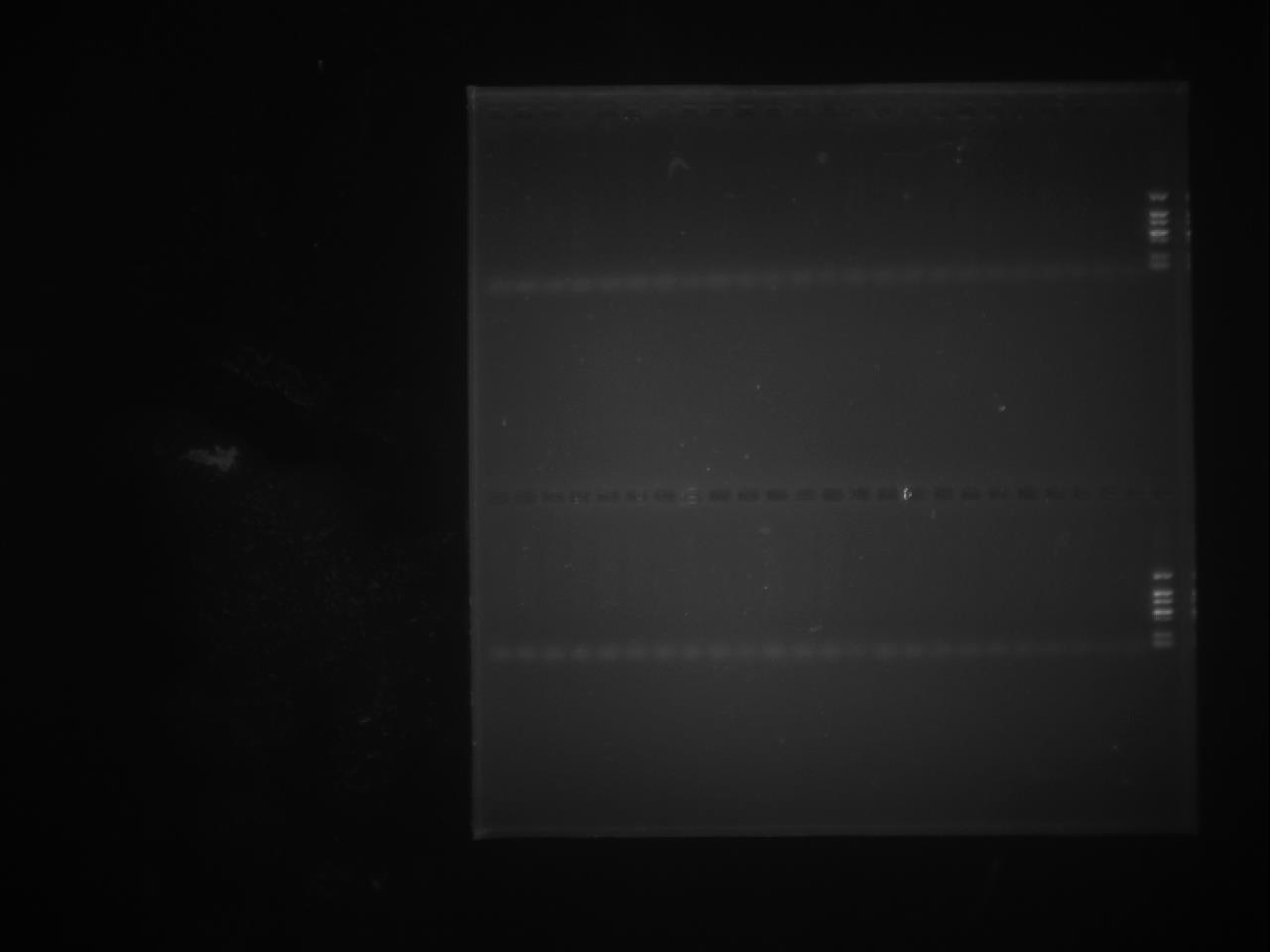

Supplement: Supplemental Information 1 [file peerj-12-17648-s001.zip › original image/Figure 5. Identification of MAT genotypes of single-conidium in degenerate strains by PCR amplification/xm-1/xm-1(c,d).jpg]
